# Searchlight Classification Informative Region Mixture Model (SCIM): Identification of Cortical Regions Showing Discriminable BOLD Patterns in Event-Related Auditory fMRI Data

**DOI:** 10.3389/fnins.2020.616906

**Published:** 2021-02-01

**Authors:** Annika Urbschat, Stefan Uppenkamp, Jörn Anemüller

**Affiliations:** Department of Medical Physics and Acoustics, Carl von Ossietzky Universität Oldenburg, Oldenburg, Germany

**Keywords:** MVPA, searchlight classification, SVM, GMM, *p*-values, fMRI

## Abstract

The investigation of abstract cognitive tasks, e.g., semantic processing of speech, requires the simultaneous use of a carefully selected stimulus design and sensitive tools for the analysis of corresponding neural activity that are comparable across different studies investigating similar research questions. Multi-voxel pattern analysis (MVPA) methods are commonly used in neuroimaging to investigate BOLD responses corresponding to neural activation associated with specific cognitive tasks. Regions of significant activation are identified by a thresholding operation during multivariate pattern analysis, the results of which are susceptible to the applied threshold value. Investigation of analysis approaches that are robust to a large extent with respect to thresholding, is thus an important goal pursued here. The present paper contributes a novel statistical analysis method for fMRI experiments, searchlight classification informative region mixture model (SCIM), that is based on the assumption that the whole brain volume can be subdivided into two groups of voxels: spatial voxel positions around which recorded BOLD activity does convey information about the present stimulus condition and those that do not. A generative statistical model is proposed that assigns a probability of being informative to each position in the brain, based on a combination of a support vector machine searchlight analysis and Gaussian mixture models. Results from an auditory fMRI study investigating cortical regions that are engaged in the semantic processing of speech indicate that the SCIM method identifies physiologically plausible brain regions as informative, similar to those from two standard methods as reference that we compare to, with two important differences. SCIM-identified regions are very robust to the choice of the threshold for significance, i.e., less “noisy,” in contrast to, e.g., the binomial test whose results in the present experiment are highly dependent on the chosen significance threshold or random permutation tests that are additionally bound to very high computational costs. In group analyses, the SCIM method identifies a physiologically plausible pre-frontal region, anterior cingulate sulcus, to be involved in semantic processing that other methods succeed to identify only in single subject analyses.

## 1. Introduction

Multi-voxel pattern analysis is a tool that has been established in functional magnetic resonance imaging (fMRI) analyses investigating acquired data obtained from cognitive studies. The approach provides multiple advantages compared to conventional univariate analyses strategies, e.g., general linear models (GLM, Friston et al., 1995) due to its' higher sensitivity (Norman et al., 2006). Information from comparably weak functional BOLD signals in single voxels are accumulated to better discriminable patterns of BOLD responses, which can increase the statistical power (Kriegeskorte et al., 2006). However, a standard for evaluation and interpretation of outcomes from theses multivariate analyses has not been established yet. Since the statistical nature of results from multivariate analyses (e.g., classification accuracies or area under the ROC curve for classification analyses) differs from those obtained by univariate analyses (e.g., z-scores, t-scores, beta-values), different statistical tests need to be applied to distinguish statistically significant results. In this paper we present the searchlight classification informative regions mixture model (SCIM) algorithm, a procedure to statistically evaluate multivariate pattern analysis (MVPA) results obtained from fMRI data that is robust against threshold choices while being less computationally expensive in comparison to commonly used random permutation tests.

To identify cortical regions that show distinguishable BOLD patterns for contrasted conditions, one method is the searchlight classification algorithm (Kriegeskorte et al., 2006). Local patterns of BOLD responses in spherically shaped spatial data subsets are evaluated in a classification analysis, resulting in three dimensional maps representing the local informational content about the contrasted conditions, with a classification performance value for each searchlight's center-voxel. To separate informative searchlight regions from those without information, classification results need to be tested for statistical significance. Different approaches have been used for evaluating classification performance results.

In some neuroimaging studies the single subjects results of secondary interest compared to group-level analyses due to the high variability across humans. In these cases one approach to extract informative regions from searchlight analyses are voxelwise *t*-tests across subjects for the classification performance against chance level (Bode and Haynes, 2009; Kahnt et al., 2010; Carlin et al., 2012). Since the number of subjects and therefore the samples per test are limited to low numbers in most studies, this approach for information-like measures was often criticized (Brodersen et al., 2013; Stelzer et al., 2013; Allefeld et al., 2016).

Under the null hypothesis that a classifier cannot find information about differences between two conditions in the BOLD data for underlying cognitive tasks, the classification of these data can be modeled as a Bernoulli trial, resulting in a binomial distribution for *n* independent tests, requiring independence of trials (Pereira et al., 2009). The binomial test was utilized by multiple fMRI studies (Oosterhof et al., 2010; Abrams et al., 2012; Akama et al., 2012, 2014). Most fMRI MVPA studies, however, compensate for the low number of trials accessible per subject with cross-validation in the classification analysis, violating the independence of trials in the analysis leading to too optimistic results in statistical evaluation with the binomial test. Random permutation test are a frequently used alternative to binomial tests (Allefeld and Haynes, 2014; Hausfeld et al., 2014; Arsenault and Buchsbaum, 2015) motivated by the few assumptions about the data they require (Pereira et al., 2009; Pereira and Botvinick, 2011; Stelzer et al., 2013; Allefeld et al., 2016). Under the assumption that data samples are independent of class labels, the null hypothesis expects the original classification performance to be drawn from a distribution derived by repetitions of classification analysis with randomly permuted class labels. The probability for the null hypothesis, respective, *p*-value is determined by the number of permutations that lead to an equally high or higher classification performance than the original analysis. However, the smallest *p*-value that can be achieved is one divided by the number of repetitions. Due to the high dimensionality of fMRI data, these test are computationally very expensive.

Instead of artificially creating a distribution of classification performance values that are obtained from classification of non-informative searchlight volumes by permutation of class labels, we propose to use the assumption that, for cognitive tasks, only specific brain regions will be involved while large cortical regions remain unaffected. The distribution of classification performance values obtained from all searchlight regions from the brain can then be decomposed into a non-informative searchlight distribution and an informative searchlight distribution with a two-component Gaussian mixture model (GMM, Dempster et al., 1977), assuming a Gaussian nature of the sub-distribution due to the high dimensionality of the searchlight numbers (about 10^5^ searchlights/voxels, respectively). In mass-univariate fMRI analyses similar approaches have been applied to decompose activated voxel distributions and non-activated voxel distribution, using, e.g., fundamental power frequency for decomposition (Everitt and Bullmore, 1999; Hartvig and Jensen, 2000; Vincent et al., 2010) or activation clusters (Penny and Friston, 2003; Kim et al., 2010; Oikonomou and Blekas, 2013). Pendse et al. (2009) used three-component Gaussian mixture models for this purpose—in addition to the non-activated and activated distribution, and they assumed a deactivated distribution with a decreased BOLD response for specific conditions. Given the non-directional nature of classification results, we propose to apply a two-component GMM for MVPA results.

A reliable and robust statistical evaluation is of increasing importance for the investigation of rather complex and abstract cognitive tasks, e.g., the semantic interpretation while listening to spoken language. We therefore show the applicability of the proposed method not only on artificial simulation data but also on data from an auditory fMRI study, investigating the differences in cortical regions involved in the processing of semantically valid speech utterances compared to an acoustic signal that is physically identical to normal speech but without any semantic content. The amount of literature covering speech processing by humans indicates the importance of this topic for research on human communication. Studies range from very fundamental tasks like voiced pitch or vowel perception (Liebenthal et al., 2005; Uppenkamp et al., 2006; Formisano et al., 2008) and speech recognition to more abstract tasks like phoneme recognition and finally semantic interpretation on a lexical word level (LoCasto et al., 2004; Handjaras et al., 2016) and sentence level (Friederici et al., 2000). However, abstract tasks require very careful study designs and more research is required to obtain reliable results to understand human communication basics.

The comparison of the proposed SCIM method to the binomial test shows a high robustness of SCIM against threshold choices, leading to similar results to those obtained by the frequently proposed permutation test. However, the computational cost is considerably reduced, allowing for an increased number of comparisons of conditions and better insights into cognitive processes.

## 2. Methods

### 2.1. Algorithm Architecture

**Figure 1:**
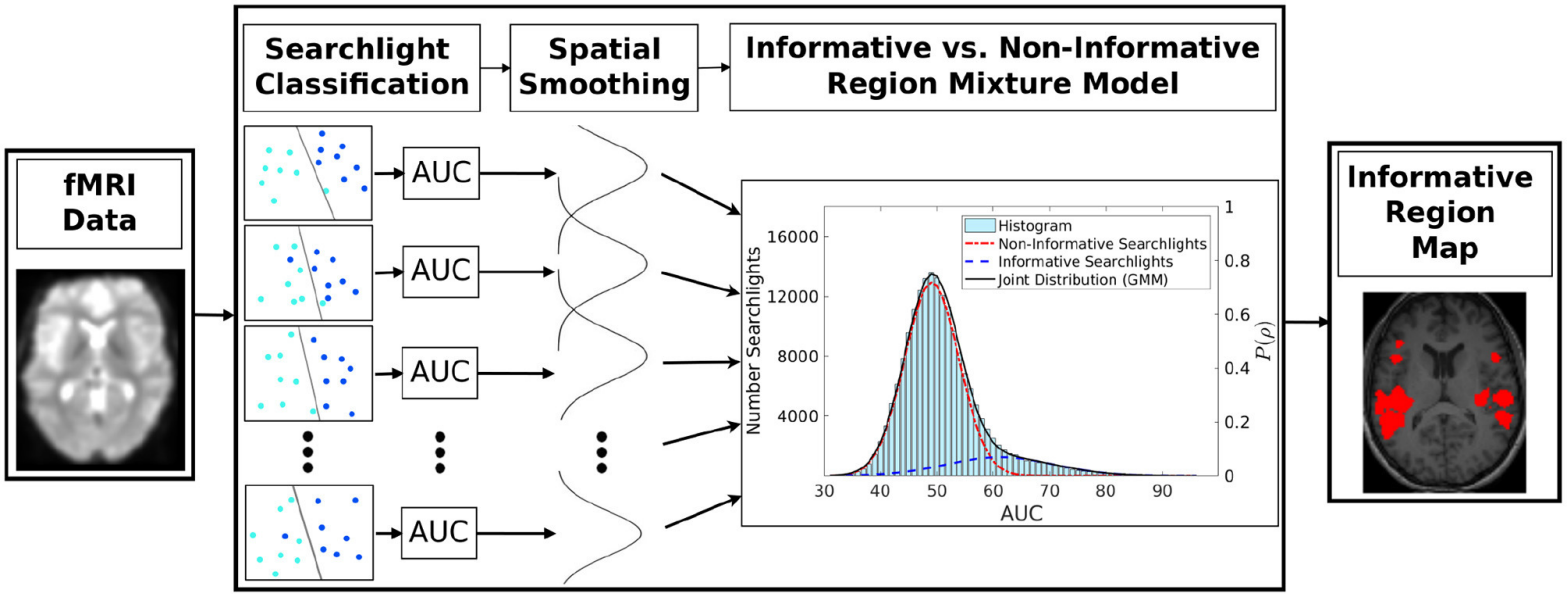
Proposed searchlight classification informative region (SCIM) algorithm procedure. Subsequent to fMRI data classification with the searchlight algorithm, the resulting area-under-curve (AUC) performance values are spatially smoothed and decomposed into a non-informative and an informative searchlight distribution using a two-component GMM. Searchlights with *a-posteriori* probability for the informative distribution above threshold, equivalent to the non-informative distribution posterior below threshold, define the informative region map (IRM).

The proposed algorithm computes for each voxel the *a-posteriori* probability of how likely it is that the small brain volume surrounding this voxel conveys information about the experimental condition. The resulting three-dimensional probability map is subsequently referred to as the informative region map (IRM). Figure 1 provides an overview of the algorithm's main steps, described in detail in subsequent sections.

*A-posteriori* probabilities are computed based on a two-component Gaussian mixture model, which models the distribution of decoding accuracy (area-under-curve values, AUC) across spatial analysis positions. Decoding is performed using searchlight classification with linear support vector machines (SVM), from which the classification accuracy for each voxel position, averaged across all stimulus presentations, is obtained.

### 2.2. Informative vs. Non-informative Region Mixture Model

We hypothesize that brain volumes, containing results from searchlight analyses, can be divided into two populations: (1) searchlights that carry information about a condition contrast and (2) searchlights that do not contain this information. Classification performance of the former is expected to be on average higher than chance level, albeit it may fluctuate considerably among informative searchlight volumes. Classification performance associated with the non-informative searchlight volumes, instead, necessarily fluctuates around chance level.

To construct a generative probabilistic model that reflects the diversity of observed classifier performance within the two groups, we adopt a two-component Gaussian mixture model where one mixture component models the non-informative searchlight distribution and the second component models the informative distribution.

**Figure 2:**
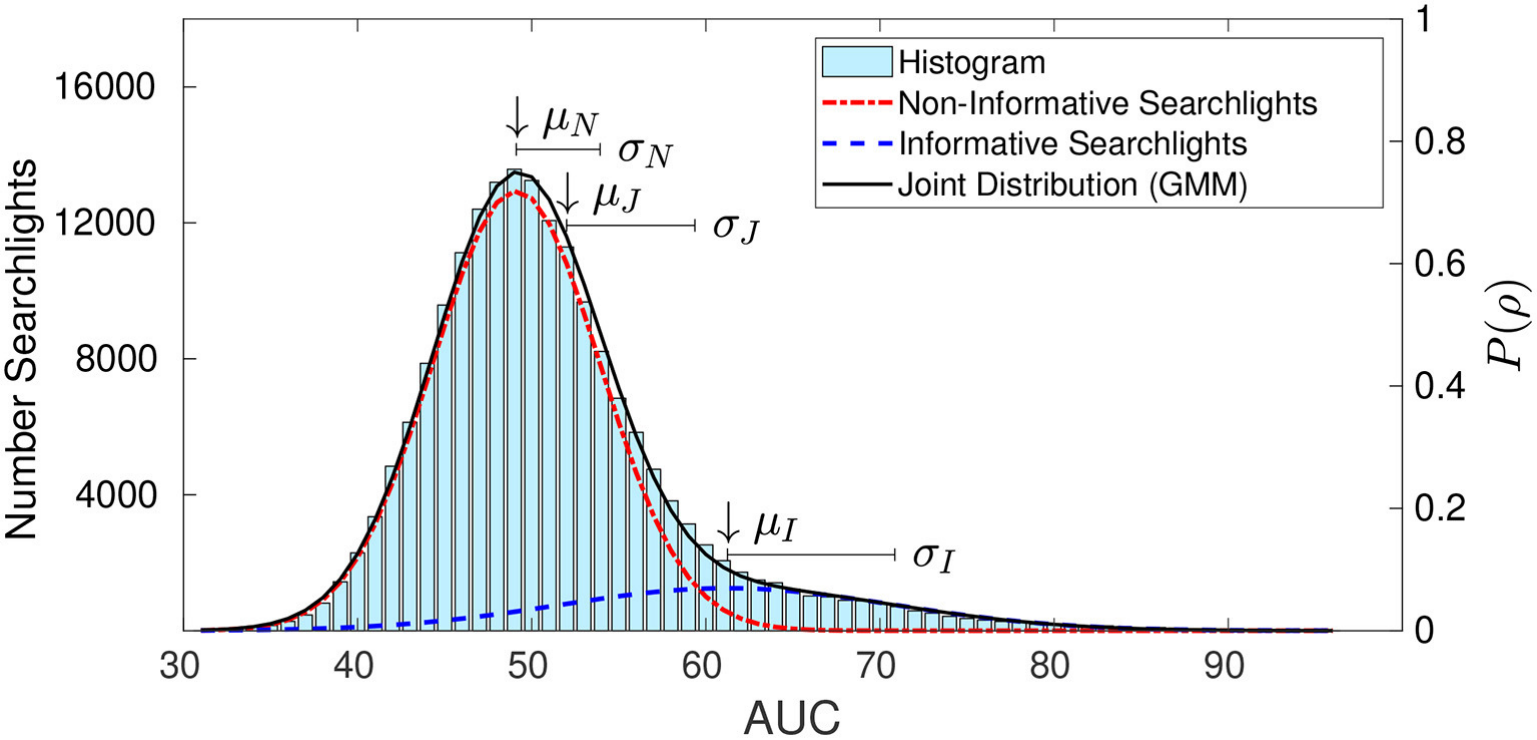
The histogram of searchlight area under the curve (AUC) values in an example map from single-subject results analysis is overlain with the respective GMM and the corresponding informative and non-informative searchlight distributions. Additionally means and variances of distributions as basis of metrics for separation criteria are displayed.

Component distributions for searchlight area-under-curve (AUC) performance values, shown in Figure 2, for the informative ($N_{I}$, blue) and non-informative ($N_{N}$, red) components are computed from the whole brain AUC histogram using the expectation-maximization (EM) algorithm (Dempster et al., 1977). The underlying mixture model links the component distributions to the joint distribution P (black) according to

(1) $$\begin{array}{l} P(\rho_{k}|\mu_{I},\mu_{N},\sigma_{I},\sigma_{N},\pi_{I},\pi_{n})=\pi_{I}N_{I}(\rho_{k})+\pi_{N}N_{N}(\rho_{k})\\ =\pi_{I}N(\rho_{k}|\mu_{I},\sigma_{I})+\pi_{N}N(\rho_{k}|\mu_{N},\sigma_{N}), \end{array}$$

where ρ_*k*_ is the classification performance AUC reached by the *k*-th SVM classifier, operating on the *k*-th searchlight volume. Estimated values of prior probabilities π_*I*_, π_*N*_, distribution means μ_*I*_, μ_*N*_, and standard deviations σ_*I*_, σ_*N*_ are obtained from subsequent iterations of expectation-step (E-step) and the maximization-step (M-step) of the EM-algorithm that maximizes the logarithmic likelihood function

(2) $$\begin{array}{l} \ln P({\left\{\rho_{k}\right\}}_{k=1}^{K}|\mu_{I},\mu_{N},\sigma_{I},\sigma_{N},\pi_{I},\pi_{n})\\ =\sum_{k=1}^{K}\ln\;\{\pi_{I}N(\rho_{k}|\mu_{I},\sigma_{I})+\pi_{N}N(\rho_{k}|\mu_{N},\sigma_{N})\}. \end{array}$$

The *a-posteriori* probability for the *k*-th searchlight to belong to the subset $C_{I}$ of informative searchlight volumes is given by,

(3) $$\begin{array}{l} p(k\text{ informative}|\rho_{k})\equiv p(C_{I}|\rho_{k})\\ =\frac{p(C_{I})p(\rho_{k}|C_{I})}{p(C_{I})p(\rho_{k}|C_{I})+p(C_{N})p(\rho_{k}|C_{N})}\\ =\frac{\pi_{I}N(\rho_{k}|\mu_{I},\sigma_{I})}{\pi_{I}N(\rho_{k}|\mu_{I},\sigma_{I})+\pi_{N}N(\rho_{k}|\mu_{N},\sigma_{N})}. \end{array}$$

Conversely, the probability of *k* being from the subset $C_{N}$ of non-informative searchlight volumes is

(4) $$\begin{array}{l} p_{\text{SCIM}}(k)\equiv p(k\text{ non-informative}|\rho_{k})=p(C_{N}|\rho_{k})\\ =\frac{\pi_{N}N(\rho_{k}|\mu_{N},\sigma_{N})}{\pi_{I}N(\rho_{k}|\mu_{I},\sigma_{I})+\pi_{N}N(\rho_{k}|\mu_{N},\sigma_{N})}\;\\ =1-p(C_{I}|\rho_{k}). \end{array}$$

The latter quantity, *p*_SCIM_, is used throughout the manuscript since it facilitates comparison with the classic *p*-value of reference methods that denotes the probability of accepting the Null-hypothesis. Thus, searchlight volumes with *p*_SCIM_ below threshold indicate informative searchlight volumes and in their entity constitute the informative region map (IRM).

### 2.3. Searchlight Classification

The searchlight algorithm requires the spatial division of the data set into overlapping, near spherically shaped searchlight volumes centered around each voxel, the center-voxel of the respective searchlight sphere, with the radius set to three voxels. BOLD activations of voxels within a particular searchlight span a multidimensional feature-space vector **x** from which the corresponding experiment condition label *y* = ±1 is to be predicted.

Subsequent to all searchlight volumes being analyzed independently of each other, classification performance results are mapped to the respective center-voxel of each searchlight, resulting in a three-dimensional information-based map (Kriegeskorte et al., 2006) that reflects the information conveyed within local BOLD regions about the experimental contrast. Information-based maps reflect the informational content in local BOLD patterns based on their separability in a high-dimensional feature space. The absolute activation strength is not important for the interpretation.

### 2.4. Support Vector Machine

The classification analysis was based on linear support vector machine analysis (SVM, Schölkopf and Smola, 2001), which is a suitable and robust classification method for fMRI data (e.g., Misaki et al., 2010). A logistic regression model in pilot experiments led to comparable but slightly lower classification performance results. SVMs are discriminative classifiers, finding a separating hyper-plane in feature space with maximum distance to the respective class-samples. The resulting model is parameterized by an optimum weight-vector w^*^ that projects data-samples **x**_*i*_ orthogonally to the separating hyper-plane. To allow for overlapping class-distributions, the soft-margin linear SVM solution is obtained by minimizing a cost function that includes the projection term ${\text{w}}^{T}{\text{x}}_{i}$ as well as a regularization term **w**^*T*^**w**, resulting in the optimum

(5) $${\text{w}}^{*}=\underset{\text{w}}{\text{argmin}}\;(\frac{1}{2}{\text{w}}^{T}\text{w}+C\sum_{i=1}^{l}\max{\left(1-y_{i}{\text{w}}^{T}x_{i},0\right)}^{2}).$$

To avoid overfitting, the regularization parameter *C* is determined from experimental data by nested cross-validation in which an inner cross-validation loop is employed to find the optimal *C* through grid-search, and an outer cross-validation loop repeatedly estimates classifier performance on held-out data.

### 2.5. Area Under the Curve (AUC) Analysis

Performance of the classifier at each searchlight's spatial position is measured as the area under the curve (AUC), a quantity that is independent of a specific classifier threshold value since it is computed by integrating the area under the receiver operating characteristic (ROC) curve of true- and false-positive rates. AUC has been shown to provide a reliable performance measure with advantageous properties in a number of classification problems, as confirmed by, e.g., Bradley (1997), and can be interpreted as the probability of a correct classifier decision in a pairwise comparison task of one positive and one negative example being drawn at random from the data ensemble (Green and Swets, 1966). In a number of analyses performed here (cf. results), the overall accuracy measure of the percentage of correct classifications is used as an alternative to AUC, to investigate the impact on the overall SCIM system's analysis results.

### 2.6. Spatial Smoothing

To decrease the effect of inter-individual anatomical differences across participants and to avoid destroying potential fine grained structure that might support classification, classification performance maps were spatially smoothed with Gaussian kernel (FWHM 3 mm) instead of a spatial smoothing step during the pre-processing as it is common in multivariate analysis procedures.

### 2.7. Metrics for Separation of Informative and Non-informative Distributions

The degree to which our hypothesis of underlying informative and non-informative voxel distributions is fulfilled can be estimated by the separation of the informative distribution (mean μ_*I*_, standard deviation σ_*I*_) and the non-informative distribution (μ_*N*_, σ_*N*_) after the two-component mixture model has been fit to the searchlight AUC performance values. The classic metric for the separation of two normal distributions is the sensitivity index, which is given by

(6) $$\begin{array}{l} d^{\prime }=\frac{\mu_{I}-\mu_{N}}{\sqrt{\frac{1}{2}\left(\sigma_{I}^{2}+\sigma_{N}^{2}\right)}}. \end{array}$$

The resulting *d*′ values are included as a model selection parameter in the cross-validation procedure for regularization.

We note that a number of separation criteria have been evaluated as alternatives to the sensitivity index, including several mean- and variance-based measures, geometric distribution overlap, and Kullback-Leibler divergence. The corresponding results showed no systematic differences to the *d*′ sensitivity index.

### 2.8. Baseline Statistical Tests

Previous studies have applied a number of different statistical tests to obtain thresholded result maps from multivariate fMRI analyses, two commonly used tests being the binomial test (Oosterhof et al., 2010; Abrams et al., 2012; Akama et al., 2012) and the random permutation test (Allefeld and Haynes, 2014; Hausfeld et al., 2014; Kumar et al., 2016).

In the binomial test, *p*-values are computed as the probability for *n* coincidentally correct classifications in *N* trials according to Equation (7), with *p*_*T*_ the a priori probability of the target class, i.e., the prior probability of a semantic speech stimulus, and *p*_*F*_ = 1 − *p*_*T*_ the probability of a non-target stimulus,

(7) $$p_{\text{bin}}(k)=\left(\begin{array}{c} N\\ n \end{array}\right)p_{T}^{n}p_{F}^{\left(N-n\right)}.$$

The random permutation test is based on repeated application of the entire classification procedure (cf. searchlight classification) on data with label-independent data. In each of *N*_*r*_ repetitions, target labels are shuffled randomly to simulate independence of samples and targets. *p*-values are subsequently obtained from the number of repetitions *n*_*h*_, that led to an equal or higher classification performance than the performance obtained from the original (unshuffled) data set, divided by the total number of repetitions *N*_*r*_,

(8) $$\begin{array}{l} p_{\text{rp}}\left(k\right)=\frac{n_{h}}{N_{r}}. \end{array}$$

A direct comparison for the two reference evaluation tests can be found in Stelzer et al. (2013).

### 2.9. Speech Stimuli

The aim of the present study is the identification of cortical structures that are engaged in semantic processing of speech. To disambiguate simultaneous physical and semantic stimulus differences that occur, e.g., when contrasting speech with noise, we employed two stimulus sets, semantic and non-semantic speech, that are characterized by largely identical acoustic properties while differing only in the presence vs. absence of semantic meaning.

Non-semantic speech utterances were taken from the “International Speech Test Signal” (ISTS, Holube et al., 2010), originally designed as a test signal for language-independent hearing aid evaluation. ISTS is constructed from speech material from six female speakers with different native languages (Arabic, English, French, German, Mandarin, and Spanish), each reading a text in her mother tongue. It has been subdivided into segments of 100–600 ms duration, that were subsequently rearranged in a pseudo-random order to form a continuous stream of speech utterances. The resulting ISTS generates the percept of nonsense speech that does not contain any semantically valid statements.

Semantic speech stimuli are sentences chosen at random from the Göttingen sentence test (Kollmeier and Wesselkamp, 1997), a speech intelligibility test comprised of phonetically balanced sentences, that each convey a short semantically valid statement. To achieve perceptual comparability to the ISTS, the sentence test's male voice was transformed to a female voice percept by pitch-shifting and digitally changing the vocal tract using the tandem straight (Kawahara and Morise, 2011) method.

### 2.10. Data Acquisition

FMRI data were recorded in a 3T Siemens MRI scanner. Nineteen subjects participated in the study (11 male, 8 female, 23.5 ± 2.6 years in age), 18 of them with German as mother tongue. The latter participants were considered for further data analysis. All subjects participated voluntarily with an expense allowance.

Subjects were presented with semantic and non-semantic speech stimuli, as described above, in a passive listening paradigm. A sparse imaging design was employed with a time of repetition (TR) of 9 s, including 6.1 s of sound presentation followed by 2.9 s of EPI sequence data acquisition of a complete brain volume in 21 slices with a voxel size of 3.125 × 3.125 × 3.9 mm, a field of view of 20 × 20 cm, a matrix size of 64 × 64 and an echo time (TE) of 55 ms. Sparse imaging allows for the separation of the presentation of auditory stimuli and the scanner noise in time. In addition, the temporal overlap of measured BOLD responses for different stimuli is decreased, which has proved to be an advantage for auditory fMRI experiments (Edmister et al., 1999; Hall et al., 1999) and is also a big advantage for fMRI classification analysis. We note that semantic and non-semantic trial conditions were interleaved with five additional acoustic stimulus conditions, whose analysis is beyond the current scope and will be reported in a subsequent publication. One session comprised 50 min, including four runs with 70 trials (10 trials per condition). A T1-weighted anatomical image was recorded for each participant to allow for localization of resulting active brain regions. Pre-processing including fMRI time series motion correction, realignment and normalization to the standard MNI brain, was performed with SPM8 software (Friston et al., 1995).

### 2.11. Simulation Data

Ten simulation data sets have been created to evaluate how accurate the proposed SCIM method and the reference methods can identify regions in a data set that has been manipulated by position information about different conditions at specific locations. These spatial locations define a template map that is compared to the result maps obtained from the different evaluation methods.

A total of 80 experimental trials, 40 each per target and non-target condition, were simulated that carried information about the experimental condition only within a spatially limited template mask area, resembling the SCIM method's informative region map from one subject. In voxel regions outside of the template mask, simulated voxel activations were generated at random from a normal distribution with voxel-wise mean and variance that was identical to mean and variance computed across all experimental fMRI data across target and non-target condition. For voxels within the template mask region, voxel-wise class-specific mean and variance values were identical to mean and variance computed across all experimental fMRI data computed separately for the target and non-target condition, respectively. Obtained normal distribution voxel activations in the template mask region were spatially smoothed with a Gaussian kernel, full-width-half-maximum 3 mm, to simulate dependencies across adjacent voxels.

### 2.12. Group Level Analysis

For all three considered analysis methods, SCIM, permutation test, and binomial test, group results are obtained by pooling classification performance values separately for every voxel-position.

For the SCIM method, classification performance results are averaged across subjects, resulting in one map that represents for each voxel the mean classification performance value. Subsequently, the distribution of averaged performance values is decomposed into a non-informative and an informative searchlight distribution, similar to single subject analysis, resulting in *a-posteriori* probabilities for the non-informative searchlight distribution that are comparable to *p*-values from other statistical evaluation methods.

Permutation test group results are calculated similar to the proposed method in Stelzer et al. (2013). Classification performance values are averaged voxelwise. For every subject a set of *r* = 100 analysis repetitions with randomized labels is performed and the classification performance results are stored in *r* separate maps per subject. In the subsequent voxelwise analysis, one random sample from the set of *r* samples per subjects is selected and the corresponding classification performance at the spatial location is averaged across subjects. This procedure is repeated 100,000 times, resulting in a Null-distribution containing 100,000 samples per voxel. The resulting *p*_*rp*_-value is calculated as the number of samples within this Null-distribution that are higher or equal to the original average classification performance divided by the number of trials (100,000).

For the binomial test, the number of correct samples per searchlight/voxel is summed voxelwise. Now the assumption for one subject that the probability for the null-hypothesis' is equal to the binomial probability for *n* correct classifications in *N* samples is adapted to the sum of all correct classifications ∑*n*_*m*_ in *M* × *N* samples with a group size of *M* subjects, with *n*_*m*_ the number of correct classifications from the data of subject *m*. The resulting *p*_*bin*_-value is determined by,

(9) $$p_{bin}=\left(\begin{array}{c} M\times N\\ \sum n_{m} \end{array}\right)p_{T}^{\sum n_{m}}p_{F}^{\left(M\times N-\sum n_{m}\right)}.$$

## 3. Results

### 3.1. Simulations

The reliability of the searchlight classification informative region mixture model (SCIM) was verified with a classification analysis of simulated data. We compared informative region maps (IRMs) obtained from the SCIM method analysis to maps from searchlight classification with subsequent binomial test, random permutation test (*n* = 100 repetitions) and to the template map that underlies the simulation data.

**Figure 3:**
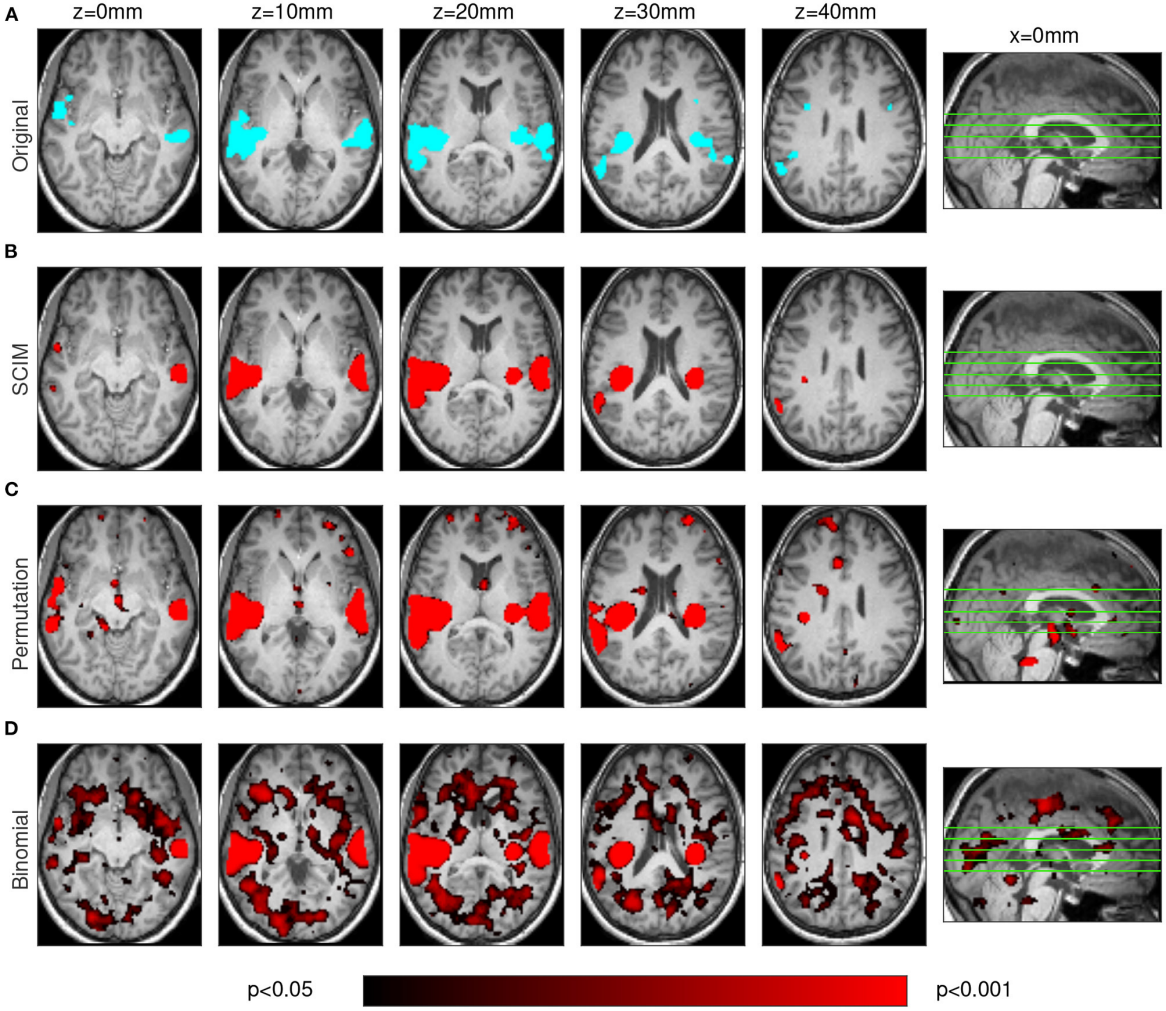
Results obtained from simulated data on smoothed AUC maps. Template map **(A)** for comparison with simulation result maps obtained from analysis with SCIM method **(B)**, random permutation test **(C)**, and binomial test **(D)** of spatially smoothed AUC maps. Due to spatial smearing effects based on the searchlight algorithm, results obtained from all methods show larger spatial extent than the template map. The map based on SCIM analysis is most similar to the template map. The map obtained from random permutation test shows larger smearing effects, while binomial test results in informative regions that are not present in the template map. The locations of the transversal slices are depicted on a sagittal slice (*x* = 0).

**Figure 4:**
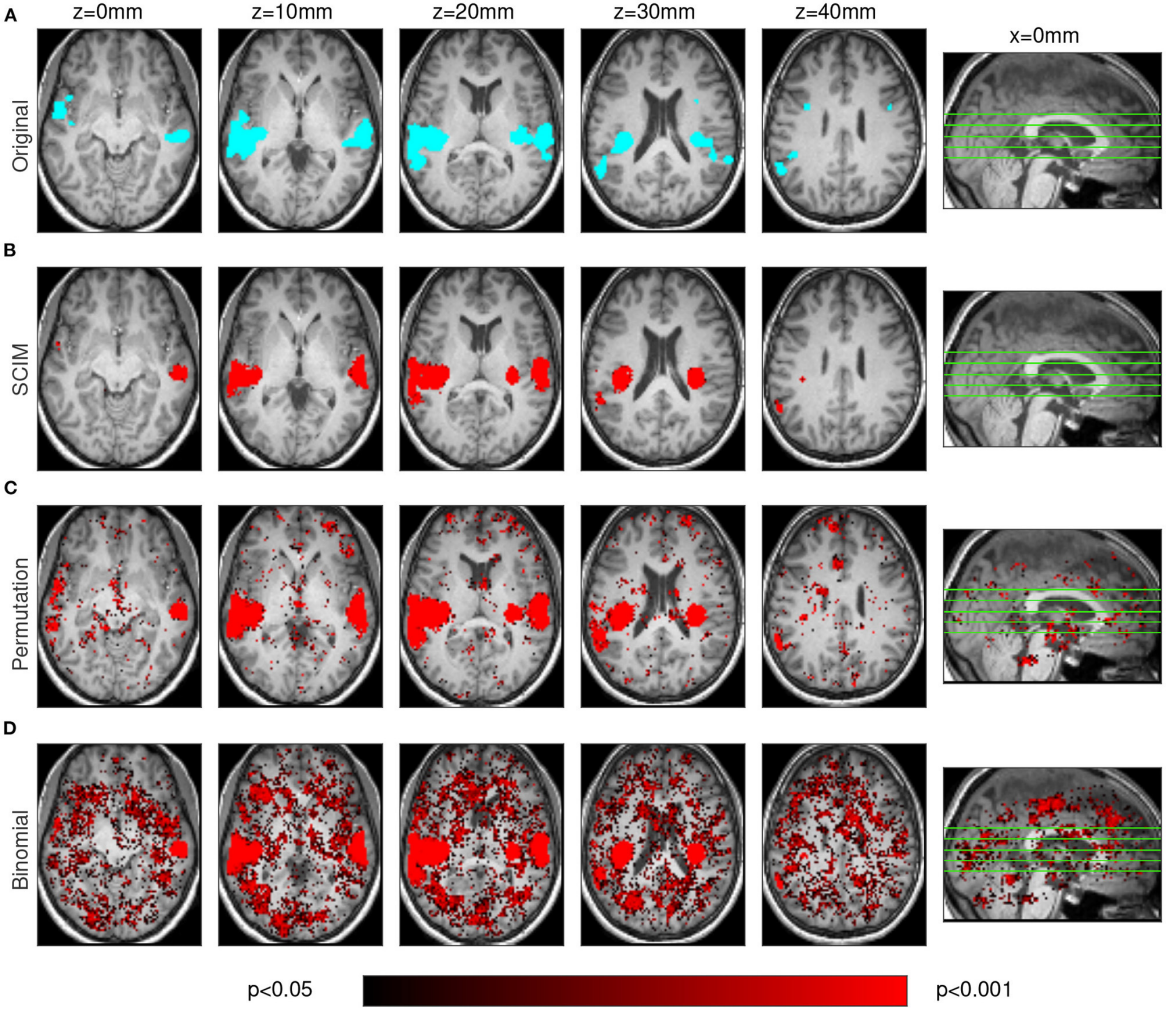
Results obtained from simulated data on unsmoothed AUC maps. The SCIM result maps **(B)** are comparable to those obtained with spatial smoothing (Figure 3) while in the permutation test results **(C)** and in the binomial test results **(D)** numerous small informative regions can be found that are not in line with the template map **(A)**.

Ten repetitions of simulation data analysis were carried out, with procedures identical to those used for experimental fMRI data, including both smoothed (Figure 3) and unsmoothed AUCs (Figure 4).

Both figures show results from one simulation run for the SCIM method, the random permutation test and the binomial test (red maps), as well as the ground-truth template map (cyan map). After spatial smoothing of the AUC maps (Figure 3), simulation data analyzed with the SCIM method and the random permutation test lead to comparable results. Informative regions obtained from these methods are slightly larger than those in the template map, which can be explained by the searchlight algorithm that spatially smears over information contained in voxels and additional spatial smoothing of AUC maps subsequent to the searchlight classification step. However, the random permutation test result map shows small additional informative regions that are not present in the template map. Results obtained with the binomial method are only valid for high significance thresholds (*p* < 0.01). For unsmoothed AUC maps (Figure 4), both reference methods, the random permutation test and the binomial test, exhibit informative regions not present in the template map. While these false positive results could be handled with cluster thresholds for the random permutation test results, the binomial test leads to invalid results. Results obtained with the SCIM method on unsmoothed AUC maps is slightly less sensitive compared to those obtained from smoothed AUC maps. Still, in comparison to the reference methods, the SCIM method best reproduces the template map.

The overlap of simulation result maps and the template map was defined as the number of voxels active in template and result maps, relative to the average total number of active voxels,

(10) $$\begin{array}{l} \text{Overlap}=\frac{n_{t\cap r}}{0.5\left(n_{t}+n_{r}\right)}, \end{array}$$

with *n*_*t*∩*r*_ the number of voxels that were active in both maps, *n*_*t*_ active voxels in the template map, and *n*_*r*_ active voxels in the result map.

**Figure 5:**
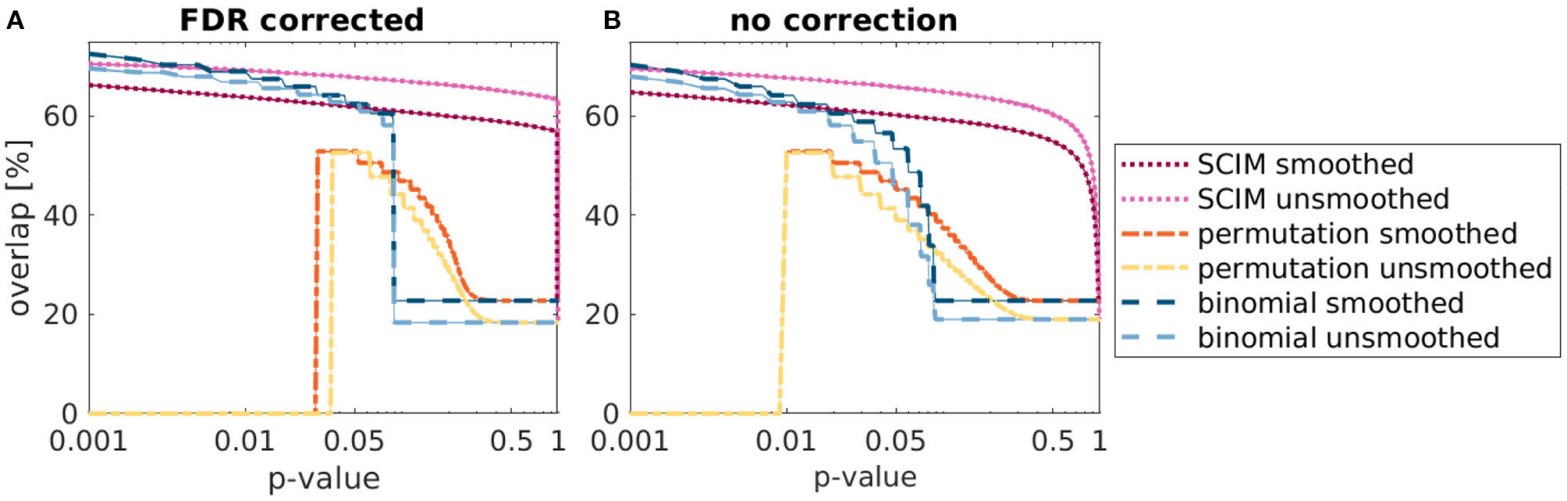
Overlap of simulation result maps with underlying ground truth map obtained from SCIM analysis, random permutation test and binomial test for 10 repetitions of simulation analysis and different applied significance *p*-value thresholds, respectively. **(A)** depicts the results with FDR correction and **(B)** depicts the results without correction for multiple comparison. Median values across 10 repetitions are presented as lines, inter-quartile ranges are displayed as semi-transparent plane but not visible due to the very small variance across repetitions. For very small *p*-values, SCIM and binomial test results show comparable overlap with ground truth maps. However, overlap decreases for binomial results with increasing *p*-values, while SCIM results stay almost constant. Result maps obtained from random permutation test show minimum *p*-values of *p*_*rp*_ = 0.01 (resulting from 100 repetitions) and exhibit no informative regions for lower *p*-value thresholds. For *p*-values higher than 0.5, both reference methods, random permutation test and binomial test, are limited by the additional criterion of *AUC*> 0.5 for searchlights to be informative and overlap values converge to a constant value. For results obtained with the SCIM method, this value is achieved for *p*-value threshold close to 1.

The statistical evaluation of simulation analysis results supports the advantage of the SCIM method compared to the reference methods. Spatial overlap of IRMs with the underlying template map for different significance thresholds is shown in Figure 5 with correction for the false discovery rate (FDR, Benjamini and Hochberg, 1995, panel A) and without correction for multiple comparisons (panel B). Medians across ten repetitions are displayed as lines and the inter-quartile range is shown as semi-transparent plane, however, not visible due to very small variance across repetitions. For low *p*-value thresholds, the overlap values are comparable for the SCIM and the binomial results, while the random permutation test results include no informative regions for very low *p*-values due to the upper limitation of resulting *p*_*rp*_-values, restricted by the number of repetitions ($p_{min}=\frac{1}{n_{rep}}$). For increasing threshold values, the overlap with results obtained by the reference methods decreases significantly, while the overlap of SCIM results with the template map stays almost constant. Even though *p*-values larger than 0.05 have little relevance in practice, the corresponding result range is shown for values up to *p*=1 in order to prove the robustness of the proposed method.

**Figure 6:**
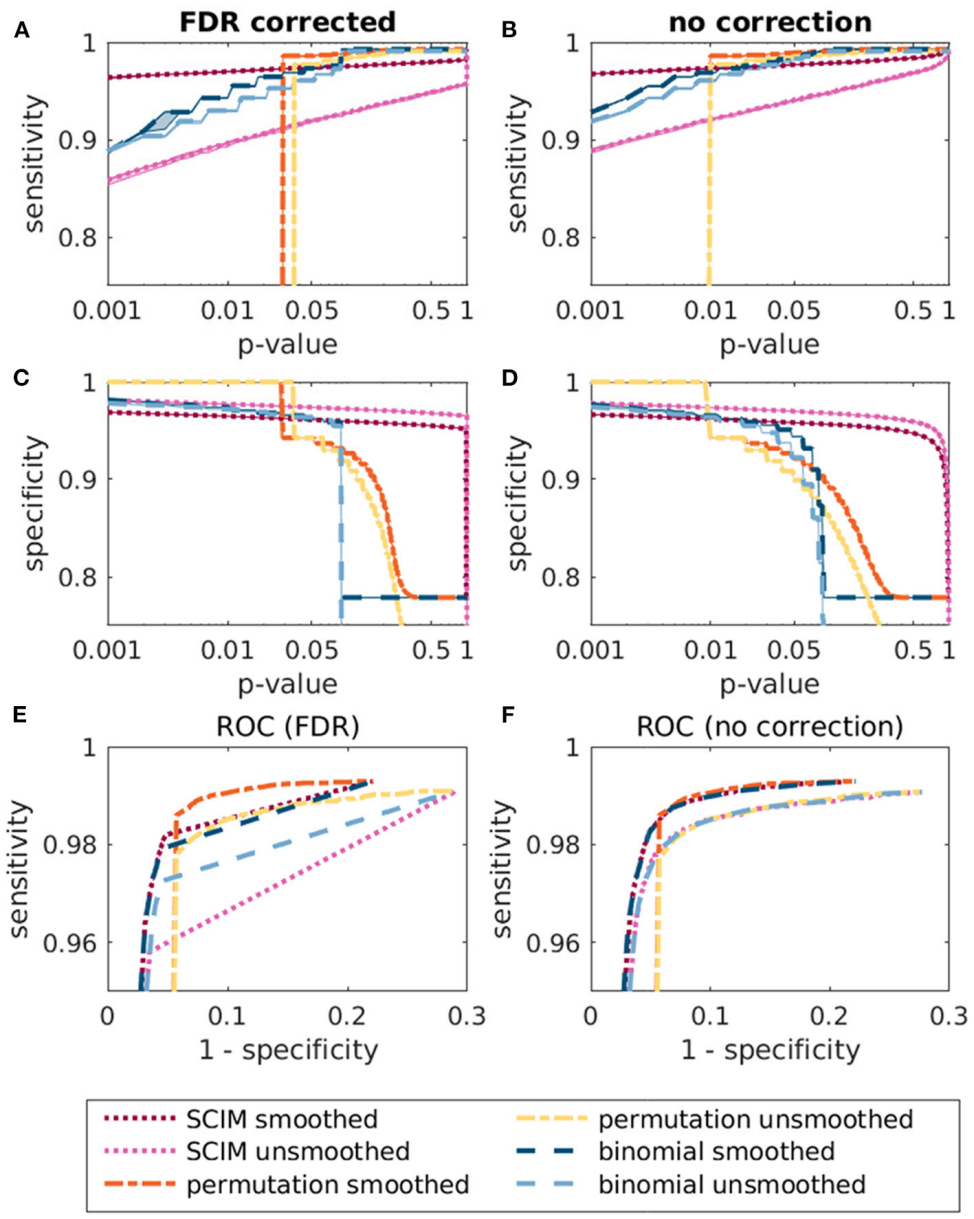
Comparison of the sensitivity **(A,B)**, specificity **(C,D)**, and ROC curves **(E,F)** for the different methods SCIM, permutation test and binomial test. All methods were tested with smoothed and unsmoothed AUC maps. The results with FDR correction are depicted in the left panels and results without correction for multiple comparison are depicted in the right panels.

The sensitivity, the specificity and the ROC curves for the different methods are depicted in Figure 6 for result maps with and without correction for multiple comparison. Except for the SCIM method with unsmoothed AUC maps, all methods reach a high sensitivity for *p*-values larger than 0.01. The difference between the specificity for smoothed and unsmoothed maps in the SCIM algorithm, however, is comparably small. The specificity of the permutation test and the binomial test decreases comparably fast for *p*-values larger than 0.01. The ROC curves for the corrected tests show an advantageous curve course of the permutation test with the smoothed AUC maps for *p*-values larger than 0.01. However, for smaller *p*-values the sensitivity of the permutation test is zero. For unsmoothed AUC maps, the curve courses can be separated in two groups, where the methods are applied to smoothed and unsmoothed maps, respectively.

### 3.2. Single Subject Results

In this section analyses of single subject results are presented. For the spatial distribution of classification analysis results, single slices from single subject results are displayed for three different participants. Quantitative analyses are performed across all subjects.

#### 3.2.1. Spatial *P*-Value Distribution

**Figure 7:**
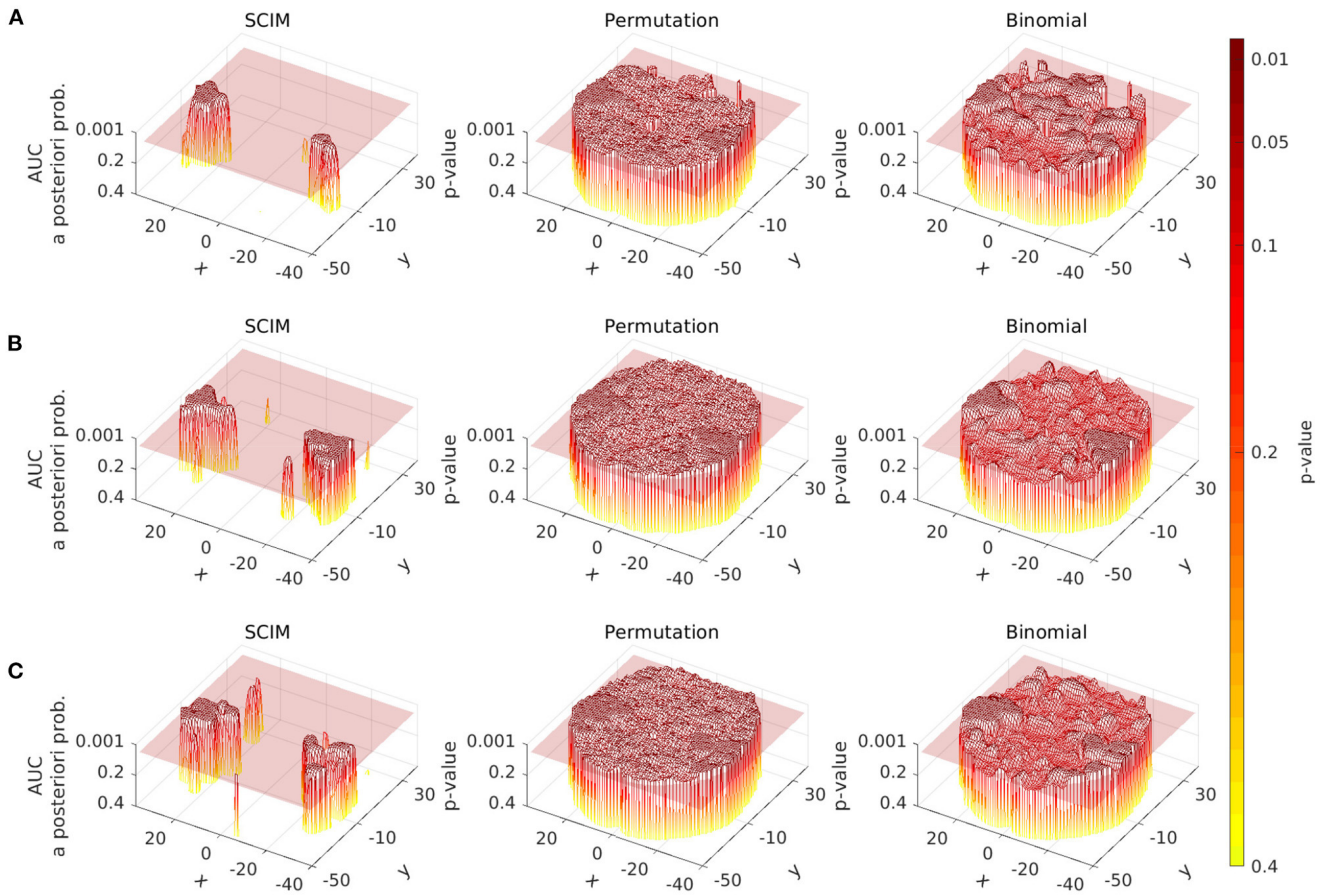
Spatial distribution of *a-posteriori* probabilities *p*_SCIM_ (SCIM) and *p*-values (random permutation test and binomial test) across a single slice (*z* = 6 mm, single subject, evaluation measure AUC, spatial smoothing for SCIM, random permutation, and binomial) of single subject results from three different subjects. **(A)** depicts the results for subject 1, **(B)** shows the results for subject 2, and **(C)** shows the results for subject 3. Left panels: distribution of *p*_SCIM_-values resulting from the Searchlight Classification Informative Region Mixture Model (SCIM, semi-transparent plane located at *p*_SCIM_ = 0.05) has plateaus of high significance levels for informative searchlight regions and a low noise floor across non-informative areas. Center panels: *p*-values resulting from random permutation analysis (semi-transparent plane located at *p*_perm_ = 0.05). Right panels: The distribution of binomial-test *p*-values (semi-transparent plane located at *p*_bin_ = 0.05) shows gradual transition from informative to non-informative searchlight areas in a narrow *p*-value interval. Differences between informative and non-informative areas are best delineated by the SCIM method and less pronounced with random permutation and binomial methods.

In Figure 7 the spatial distribution of *a-posteriori* probabilities from the SCIM analysis and *p*-values from the random permutation test and the binomial test are displayed for a single slice (at *z* = 6 mm) for three single subject results. Transparent slices are located at *p* = 0.05, separating informative from non-informative searchlights for non-corrected analyses. The SCIM analysis provides plateaus of high significance levels for all displayed single subject results, while non-informative searchlights correspond to regions of significance levels larger than *p* = 0.4, that are not displayed in the plots. The described effects below are true for all slices and all subjects, the plots are limited to one slice of three subjects, respectively, due to space limitations.

Informative regions obtained from permutation tests are predominantly similar to those obtained from the SCIM analysis, with the exception of isolated small informative regions that occur in the permutation test results. However, significance levels for informative and non-informative regions are not as clearly separated as in the SCIM analysis and the presence of the previously mentioned small informative regions is dependent on the applied significance thresholds.

The distribution of *p*-values resulting from the binomial test analysis shows a gradual transition from informative to non-informative searchlight areas in a narrow interval of *p*-values. This leads to high dependence of informative regions on the applied significance threshold.

#### 3.2.2. Single Subject Informative Region Maps

**Figure 8:**
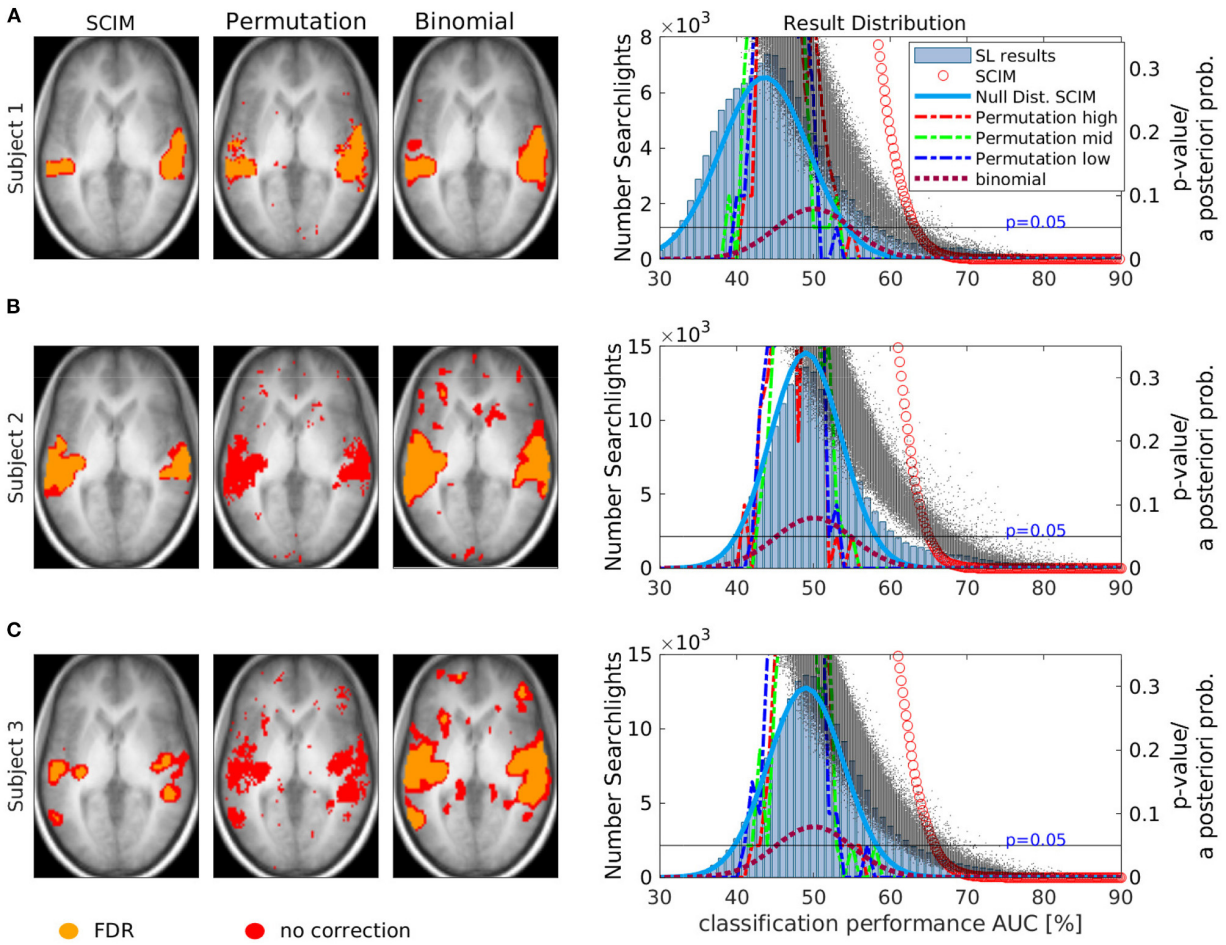
Single slice (at *z* = 6 mm) of subject informative region maps (IRMs). **(A)** depicts the results for subject 1, **(B)** shows the results for subject 2, and **(C)** shows the results for subject 3. IRMs obtained from single subject results with SCIM methods (first column), Permutation test (second column) and binomial test(third column) for three different subjects at a significance level *p*_*SCIM*_ < 0.05, *p*_*rp*_ < 0.05, and *p*_*bin*_ < 0.05, without correction for multiple comparison (red) and with FDR correction (orange), respectively. For subjects 2 and 3, no informative voxels can be found when FDR correction is applied. For the SCIM method and binomial method, maps obtained with FDR correction exhibit slightly smaller informative regions. Right panels show the results' distribution across voxels. A histogram of AUC values is presented as bar-plot. *A-posteriori* probabilities obtained from SCIM analysis (red circles) and *p*-values obtained from permutation test (black dots) and binomial test (dark red dashed line) are displayed, as well as underlying assumed Null-distributions for the different tests, SCIM method (dark blue line) and permutation test with one distribution for a voxel with high (blue), middle (green), and low (red) performance, respectively. For subject 1 the distribution peaks for AUC values lower than 50%, while for the other subjects the maximum is located at chance level AUC = 50%.

Single slices (at *z* = 6 mm) of IRMs obtained from single subject analyses and the corresponding statistical distribution of results in the whole result map are displayed in Figure 8. Result maps obtained with the SCIM method, the random permutation test and the binomial test were thresholded at *p* < 0.05, respectively. Informative regions obtained without correction for multiple comparisons are colored in red, the corresponding informative regions with FDR correction are colored in orange. Permutation test results show no informative regions after FDR correction for subjects 2 and 3, while with other methods (and for subject 1 also with random permutation) FDR correction leads to slightly decreased sizes of informative regions. Anatomical regions identified to be engaged in the semantic processing task are qualitatively similar for all methods. The right panels of Figure 8 show the histograms of classification performance values across all brain-searchlights, the assumed Null-distribution for the SCIM method (blue line) and the corresponding *a-posteriori* probabilities for classification performance values obtained with the SCIM method. Black dots show *p*-values obtained from random permutation tests, and the dashed lines show the Null-distributions for one voxel with high (red), mid (green) and low (blue) classification performance, respectively. Null-distributions obtained from the random permutation test are comparable for all three subjects, while the original distribution of AUC values is different. AUC maps resulting from the analysis of data from Subject 1 (A) are shifted toward lower values, maxima of distributions from Subject 2 (B) and Subject 3 (C) are located at chance level (*p*_*chance*_ = 50%). Only searchlights with *AUC* > 50% are considered to be informative, and less searchlights satisfying this criterion involve less comparisons to be corrected for in the FDR procedure. Therefore, the FDR corrected IRM for Subject 1 is comparable to the uncorrected one, while IRMs for Subject 2 and 3, with more searchlights being associated to the distribution of searchlights with *AUC* > 50% show no informative regions after FDR correction. The dark red dotted lines show the binomial distribution resulting from study design with *N* = 80 samples and *p*_*chance*_ = 0.5. This distribution also shows *p*-values for accomplished classification performance results as well as the assumed Null-distribution for the binomial test.

#### 3.2.3. Influence of Significance Threshold

**Figure 9:**
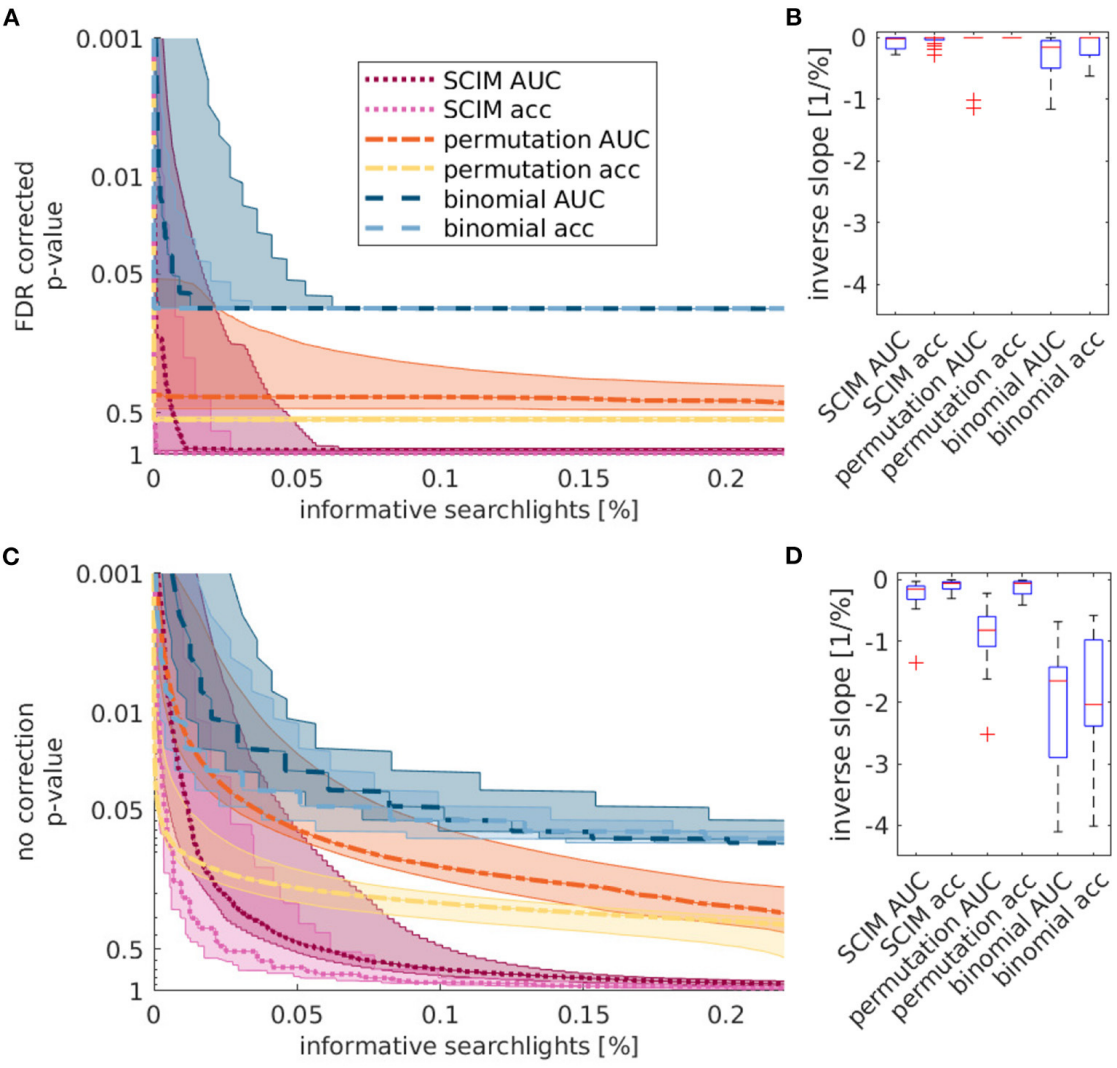
Quantitative analysis of single subject maps across subjects. **(A,C)** Cumulative histograms of fraction on searchlight volumes (in %, abscissa) whose *p*_SCIM_-value is below a chosen threshold *p*_thr_-value (ordinate), i.e., which are considered informative. Curves indicate group median for SCIM method, random permutation method and binomial method with area-under-curve (AUC) and accuracy (acc) measures, respectively. Semi-transparent areas depict the inter-quartile range. **(B,D)** Average inverse slopes of curves in **(A,C)** within the interval 0.05 > *p*_thr_ > 0.01. **(A,B)** Show FDR corrected results, **(C,D)** show respective non-corrected results. Results indicate that the SCIM method is characterized by a strong separation of informative and non-informative searchlight volumes, both for FDR corrected and non-corrected maps, while results obtained with AUC measurement and random permutation test are highly dependent on the applied thresholds. Binomial test results show this dependency in all cases.

Quantitative analyses of single subject results are displayed in Figure 9. The left panels show the portion of informative searchlights from all brain searchlights for different applied significance thresholds for the three evaluation methods, (1) SCIM, (2) random permutation, and (3) binomial test and classification performance measures, AUC and accuracy. Lines represent the median across subjects, while semi-transparent areas display the inter-quartile range. FDR corrected SCIM analysis results are approximately constant up to threshold levels of *p* = 0.1. For higher thresholds the number of informative searchlights increases only marginally. The number of informative searchlights obtained from the random permutation tests differ across subjects. For higher thresholds than *p* = 0.05 the upper quartile of the group shows a strongly increasing number of informative searchlights with increasing *p*-value thresholds, while the median of the group shows an almost constant trend, similar to the results obtained with the SCIM method.

Since searchlights that lead to very low classification performance results are associated with low *p*-values but are not expected to be highly informational, only searchlights with higher classification performance than 50% are considered to be informative. This additional criterion is the limiting factor for thresholds around *p*_*rp*_ = 0.5 for the random permutation test and *p*_*bin*_ = 0.08 for the binomial test. For higher significance thresholds all searchlights with a higher performance value than 50% are considered to be informative, irrespective of the exact applied *p*-value thresholds.

Corresponding statistics for non-corrected maps in the lower left panel show strong dependencies on applied *p*-value thresholds for both performance measures, AUC and accuracy for the binomial test, and AUC measure for the random permutation test. SCIM method results and random permutation results obtained from the accuracy analysis are nearly constant up to *p*-values of 0.05. The number of informative searchlights obtained from the SCIM analysis increases slowly for higher thresholds, while IRMs obtained from the random permutation analysis show a sharply increasing number of searchlights with increasing *p*-value thresholds.

Usual *p*-value thresholds vary between *p* = 0.01 and *p* = 0.05 across studies. The impact of different applied thresholds for significance is represented in the right panels (B and D) of Figure 9 as the inverse slope of median curves from panels A and C in the range between *p* = 0.01 and *p* = 0.05, respectively. The red lines show the median, the boxes the inter-quartile range, the dashed lines 5- and 95%-quantiles and the red crosses show outliers. For FDR corrected maps (panel B), the SCIM results show very low differences in this range. Random permutation tests lead to no informative searchlight with FDR correction, except for two subjects, that show larger differences in the mentioned range than SCIM results. Binomial tests show larger differences across thresholds than both, SCIM and random permutation results. Without correction for multiple comparisons (panel D) inverse slopes for SCIM results only differ marginally from those obtained with FDR correction. For the random permutation test on AUC values, the absolute value of the inverse slope and therefore the change of numbers of informative searchlights is significantly larger than for the SCIM analysis. Results obtained with the accuracy measure are comparable for SCIM analysis and random permutation tests. Binomial test results lead to significantly larger absolute values of inverse slopes for both, AUC and accuracy measure.

### 3.3. Group Level Results

#### 3.3.1. Summary of Evaluation Methods and Classification Measures

**Figure 10:**
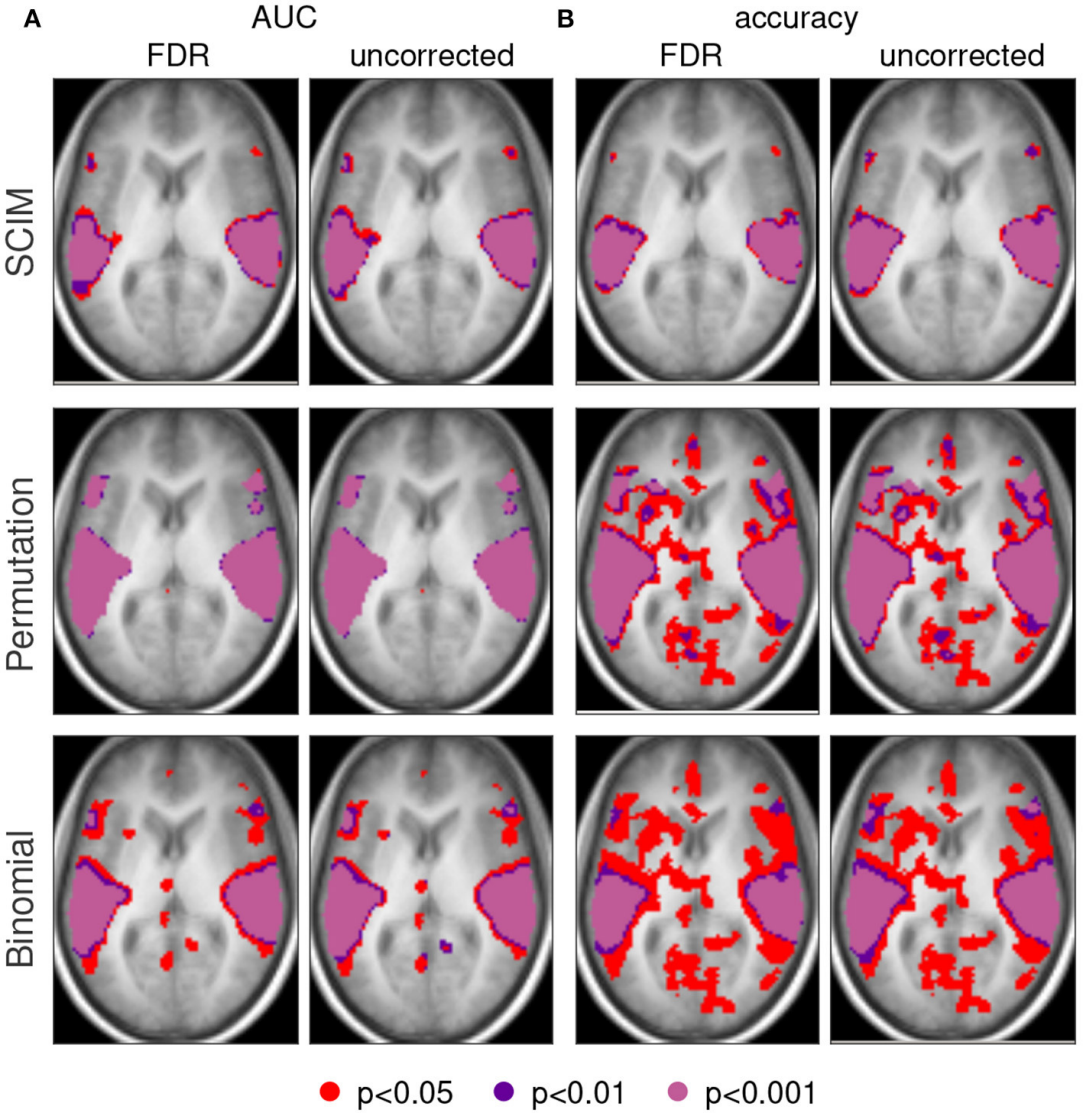
Single slices (*z* = 6 mm, respectively) of group maps with AUC measure **(A)** and accuracy measures **(B)**. First and third column show results with FDR correction, second and fourth column respective results without correction for multiple comparison. Group results obtained with the proposed SCIM methods are displayed in the first row, random permutation test results in the second row. Third row represents results from binomial test group results. Informative regions at a threshold with *p* < 0.05 are colored in red, respective results for thresholds *p* < 0.01 in dark violet and *p* < 0.01 in light violet.

Figure 10 shows the group level informative region maps (IRMs) obtained with the three different approaches SCIM method, random permutation test, and binomial test. Informative regions obtained with an applied *p*-value threshold of *p* < 0.05 are colored in red, respective informative regions for *p* < 0.01 in dark violet and *p* < 0.001 in pale violet. For AUC measures (panel A) the informative regions do not differ considerably for the different applied thresholds and with or without FDR correction when significance evaluation is performed with SCIM or random permutation test. Binomial test results show additional informative regions to those obtained with the previous two methods in anatomical areas that do not overlap with results known from literature for the investigated cognitive task, both with and without FDR correction. However, informative regions obtained with the binomial test methods with significance thresholds lower than *p* = 0.01 are similar to those obtained with the two other methods. For accuracy measures (panel B), the IRMs obtained with the random permutation test and the binomial test for *p*-value thresholds at *p* < 0.05 include informative regions in anatomical areas that are not consistent with areas known from the literature. Informative regions obtained with thresholds *p* < 0.01 are consistent with those obtained from AUC analysis and SCIM analysis.

#### 3.3.2. Group Level *P*-Value Distribution

**Figure 11:**
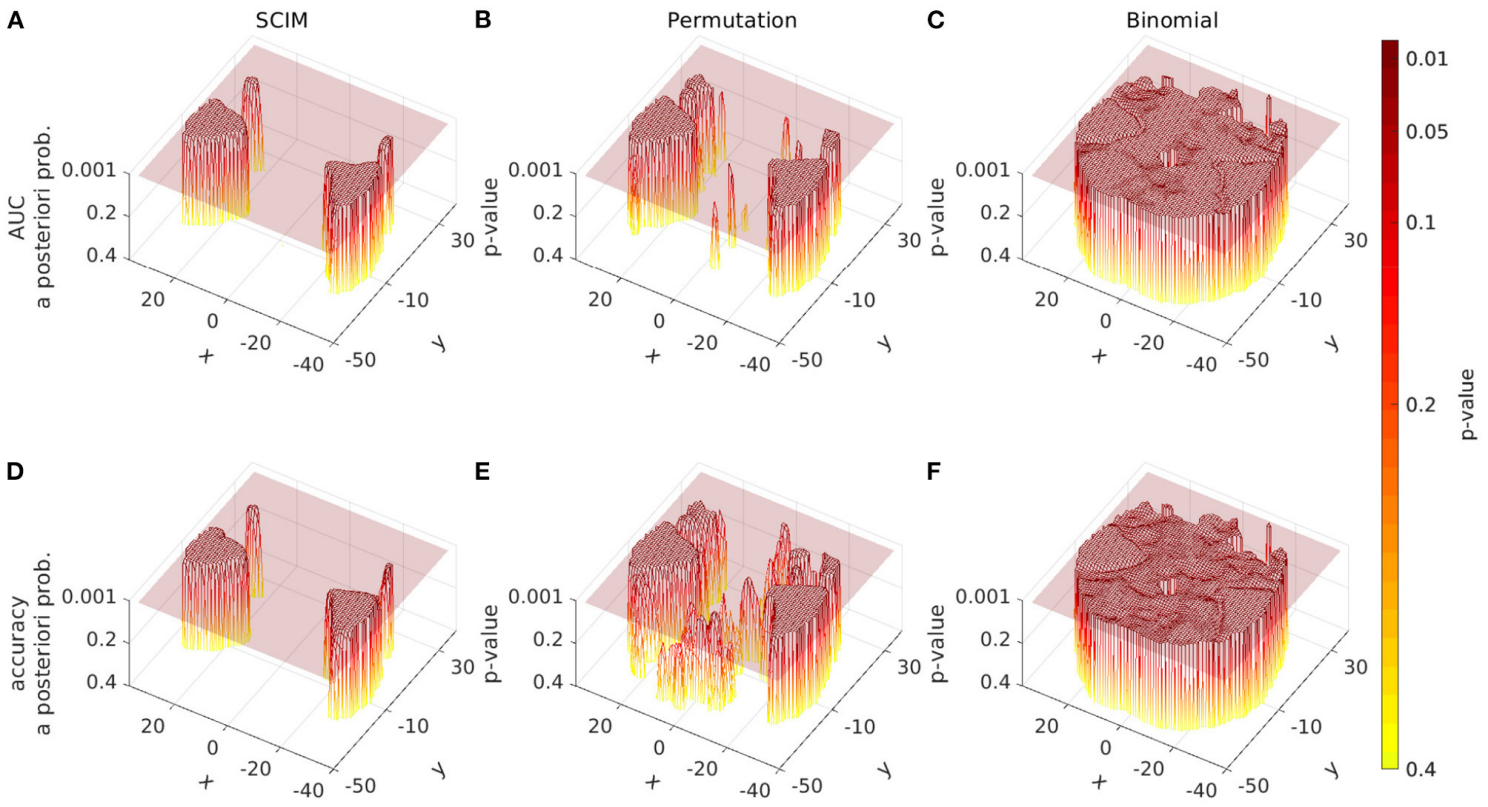
Spatial distribution of *a-posteriori* probabilities *p*_SCIM_ (SCIM) and *p*-values (random permutation test and binomial test) across a single slice from group result maps [*z* = 6 mm, group results, evaluation measure AUC **(A–C)** and accuracy **(D–F)**, spatial smoothing]. **(A,D)** Distribution of *p*_SCIM_-values resulting from the Searchlight Classification Informative Region Mixture Model (SCIM, semi-transparent plane located at *p*_SCIM_ = 0.001) has plateaus of high significance levels for informative searchlight regions and a low noise floor across non-informative areas. **(B,E)** *P*-values resulting from random permutation analysis (semi-transparent plane located at *p*_perm_ = 0.001). **(C,F)** Distribution of binomial-test *p*-values (semi-transparent plane located at *p*_bin_ = 0.001) in a very narrow *p*-value interval. Differences between informative and non-informative areas are best delineated by the SCIM method, however, very similar to those in results obtained from random permutation test. For accuracy measure, the random permutation test exhibits sub-threshold non-informative regions, that are not as well-separated from informative regions as compared to results map from AUC analysis or SCIM analysis. Results obtained from the binomial method are almost non-separable into informative and non-informative regions, since the range of emerging *p*-values is very small.

The spatial distribution of *a-posteriori* probabilities *p*_*SCIM*_ obtained from the SCIM analysis and *p*_*rp*_-values from the random permutation test as well as *p*_*bin*_-values from the binomial test are displayed for a single slice (*z* = 6 mm) of group result maps in Figure 11. Other slices show similar effects, but are not shown here due to space restrictions. The semi-transparent slices are located at thresholds of *p* = 0.001. Informative regions, i.e., the segments above a semi-transparent plane, are comparable for results obtained from SCIM and the random permutation test. However, the random permutation test on accuracy measures leads to just below threshold results that are not as clearly separated from informative regions as compared to results obtained from AUC measures or the SCIM analysis on both measures, AUC and accuracy. The *p*_*bin*_-values obtained from the binomial test lie in a very small value range that does not permit a reliable separation of informative from non-informative regions. While the other methods, SCIM and random permutation test, show spatially smooth plateaus of high significance (respectively low *p*-values), binomial test results show very homogeneous spatial distributions for both, AUC and accuracy measure.

#### 3.3.3. Statistical Distribution of Group Level Results

**Figure 12:**
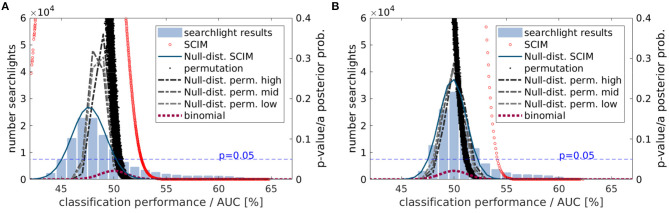
Statistical evaluation of group results based on **(A)** AUC measures and **(B)** accuracy measures. Histograms show the distribution of classification performance results emerging in group mean maps. Red circles show *a-posteriori* probabilities obtained from SCIM analysis for respective classification performance values and the blue line the underlying Null-distribution. In random permutation test *p*-values are calculated independently for all voxels that are shown with black dots. *p*-values obtained with the binomial test result from the binomial distribution that also represents the assumed Null-distribution for this test.

The distribution of group results obtained with the different approaches can be found in Figure 12 for the AUC measure (A) and the accuracy measure (B). Light blue bars display the histogram of the respective average classification performance results across all subjects. Red circles show the corresponding *a-posteriori* probabilities obtained with the SCIM method, with less data points for the accuracy measure due to the limited resolution of 80 samples per searchlight. The underlying assumed Null-distributions for the SCIM analyses are shown as dark blue lines. The *p*_*rp*_-values resulting from the random permutation tests are displayed as black dots. In comparison to single subject results (cf. Figure 8D), group result random permutation *p*_*rp*_-values show less variance for respective performance measures. However, low *p*_*rp*_-values are associated with lower classification performance values compared to *a-posteriori* probabilities (*p*_*SCIM*_) obtained from SCIM analyses. For the binomial test, the assumed distribution is equal to the resulting *p*_*bin*_-values for respective classification performance values. These are displayed as dashed dark red lines. For the AUC measure, the assumed Null-distributions from SCIM analyses and the random permutation analyses do not peak at the expected chance level at *p*_*chance*_ = 0.5 in contrast to the assumed Null-distribution for accuracy measure. However, while the SCIM Null-distribution is determined by the histogram of AUC values the Null-distribution resulting from random permutation tests does not follow the histogram of achieved AUC values. The binomial Null-distribution, on the other hand, that only depends on the study design but not on analysis results, is distributed around the expected chance level. For the accuracy measure, all Null-distributions are centered around chance level. For both measures, AUC and accuracy, the SCIM analysis provides a more stringent selector for informative regions in classification performance maps than the random permutation test and the binomial test.

#### 3.3.4. Group Level Informative Region Maps

**Figure 13:**
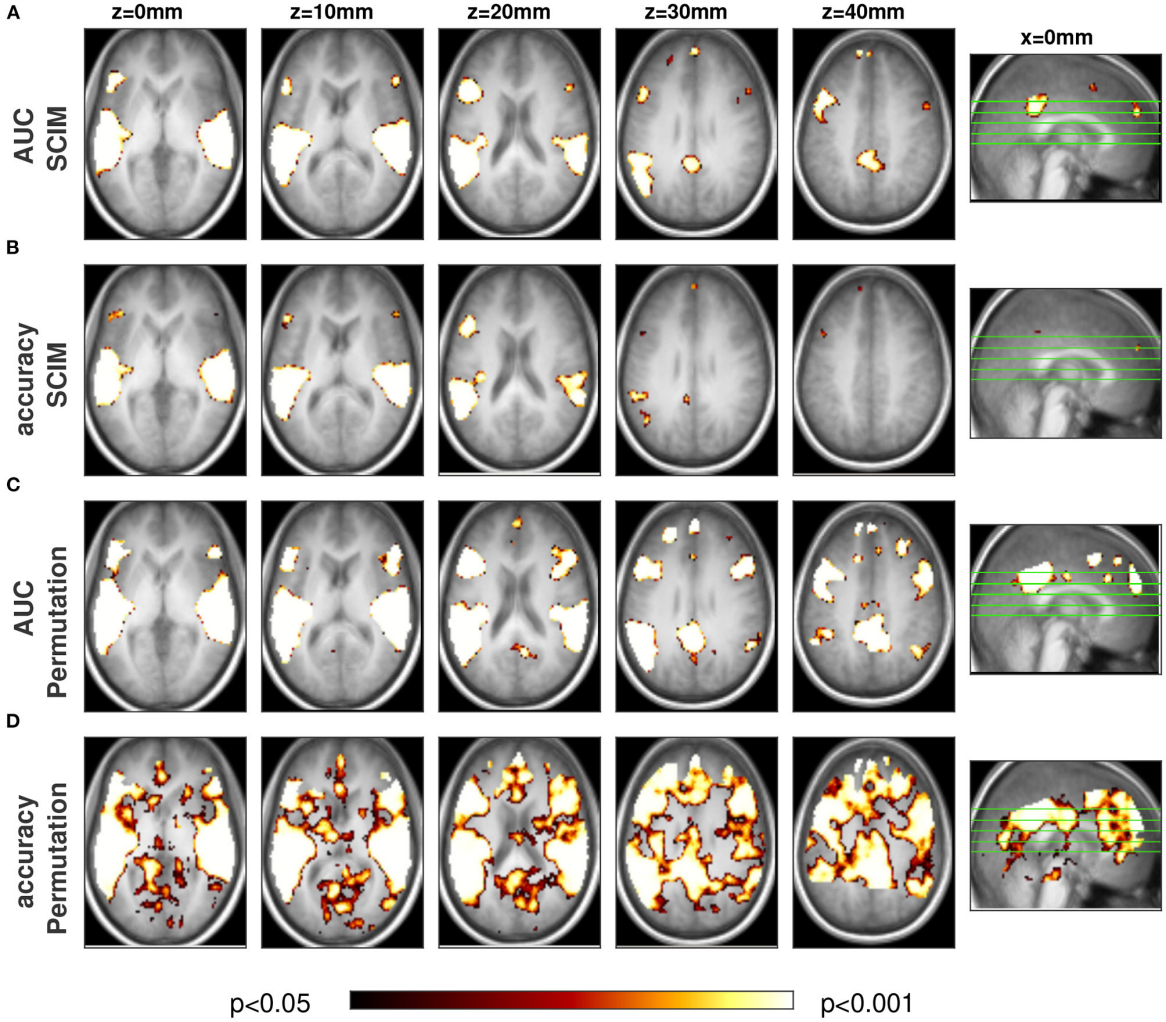
Group result maps for the contrast semantic speech vs. non-semantic speech, with proposed SCIM method on **(A)** AUC maps, **(B)** accuracy maps, random permutation test on **(C)** AUC and **(D)** accuracy maps in five transversal slices and one sagittal slice to display location of transversal slices. Informative regions for group results obtained with random permutation test and SCIM method on AUC maps are qualitatively consistent, however spatial extent of informative regions from random permutation test is slightly larger compared to those obtained from SCIM method analysis. While SCIM result maps based on accuracy measure show spatially smaller informative regions with less reliability, corresponding maps obtained from random permutation test seem to be too optimistic and lead to non-interpretable informative regions. For AUC measures informative regions are located in primary and secondary auditory cortex, namely in Heschl's gyrus (HG) and superior temporal gyrus (sts) as well as adjacent regions, Broca's area and Wernicke's area, that have been associated previously with speech processing. Additional informative regions can be found outside of temporal cortex, in anterior and posterior cingulate gyrus, previously being associated to semantic processing.

In Figure 13 informative regions are displayed for the contrast semantic speech vs. non-semantic speech, emerging from fMRI analysis with the proposed SCIM method based on AUC measure (A), on accuracy measure (B) and the corresponding maps obtained from commonly used random permutation test (C and D).

Informative regions arising from the proposed method (SCIM) overlap with those arising from random permutation test with AUC measure to a large extent, with slightly larger informative regions in the random permutation test results. The location of informative regions obtained from the described analyses are in primary auditory and adjacent regions ins Heschl's gyrus and the superior temporal gyrus, in Broca's area in the inferior frontal gyrus region and posterior to the auditory cortex in Wernicke's area. Additionally informative regions for semantic processing have been found in fronto-cortical regions in anterior cingulate gyrus.

The previous statistical evaluation has shown that the statistical power of the binomial test is considerably lower than the power of the SCIM method and the permutation test. Therefore, and in order to be able to display several slices of the real data within a reasonable amount of space, the result maps are focused on those obtained from the SCIM method and the permutation test. The corresponding result maps obtained from the binomial test can be found in the Supplementary Material.

## 4. Discussion

This paper presents a novel method for the evaluation of results obtained from multivariate searchlight classification analysis of fMRI data. Simulation data and data from a real auditory fMRI experiment are analyzed with the proposed SCIM method and results are compared to those obtained from two references methods, the random permutation test and the binomial test. The evaluation and comparison of the methods is based on the spatial distribution of obtained *p*-values, robustness of results for different significance thresholds and classification measures (AUC and accuracy) and consistency with results described in previous studies investigating semantic processing of acoustic stimuli.

### 4.1. SCIM Method for *a-posteriori* Probability Estimation

The analyses of simulation data in section 3.1 confirm the general applicability of the SCIM method and advantages over reference methods, random permutation test and binomial test, when results are compared to ground-truth. All methods reproduce the template map for low *p*-values with minor differences depending on certain processing stages, in particular spatial smoothing (cf. Figure 5). Without spatial smoothing, the reference methods exhibit false positive informative regions, not included in the template map, while the SCIM method shows largest consistency with the template map for both, smoothed and unsmoothed AUC maps with lower sensitivity but higher specificity with unsmoothed maps. As reflected in Figure 6 the SCIM method has a low sensitivity when it is used without spatial smoothing of the AUC maps and even with spatial smoothing the sensitivity of the reference methods is higher for *p*-values than 0.01. However, these differences are very small in comparison to the differences in specificity for *p*-values > 0.01, where the SCIM method outperform the reference methods.

For the experimental data, all three methods successfully identify informative regions, as reflected in low *a-posteriori* probabilities (*p*_SCIM_) for the SCIM method, and low *p*_rp_- and *p*_bin_-values for the random permutation and binomial methods, respectively. Robustness of the spatial extent of informative region maps (IRMs) with respect to the threshold value applied during analysis, however, is found to be dependent on the choice of analysis method. While the spatial map obtained with a binomial test is highly dependent on the applied threshold and leads to non-plausible results for increased *p*-value thresholds (lower significance levels), the random permutation analysis test is more robust than the binomial test at the price of very high computational cost. IRMs obtained with the SCIM method are characterized by spatially smooth, low *p*_SCIM_-values in informative regions that are clearly separated from non-informative, high *p*_SCIM_-regions (cf. Figure 7), and this partitioning is largely independent of the chosen threshold value. The same effect is visible for group results, as presented in Figures 13, 11, when comparing SCIM results with random permutation test. For AUC measure, *a-posteriori* probabilities obtained from group analysis with the SCIM method are comparable to those obtained from the random permutation test, though computationally of immensely higher efficiency. For the accuracy measure, the random permutation test is not only involved with high computational costs but leads also to non-interpretable results—in contrast to SCIM results, that are similar to those obtained with AUC measure, however with slightly smaller informative regions.

The dependence on applied thresholds was quantitatively illustrated in Figure 9. The number of informative searchlights in IRMs is almost constant for *a-posteriori* probabilities smaller than 0.1. In binomial test result maps, the number of informative searchlights increases in the range of typically used thresholds between *p* = 0.01 and *p* = 0.05. The same effect can be observed for random permutation result maps based on the AUC measure without FDR correction. These results emphasize the need for false discovery correction in result maps arising from random permutation and binomial tests. Shifted thresholds have a small impact on resulting IRM informative areas. In most of the quantitative comparisons of the SCIM method with the reference methods in this paper a value range up to *p* = 1 is depicted, even though values of *p* > 0.05 have little relevance for the experiment data. This was done to illustrate the robustness of the SCIM method. Given the robustness of spatial patterns for large values of *p* > 0.05, it is reasonable to expect highly robust results for lower choices of *p*-values (or, more specifically: for lower chosen *a-posteriori* probability levels). Therefore, the SCIM method might provide a tool for fMRI analysis that to some degree maintains sensitivity with increased specificity (Lieberman and Cunningham, 2009).

Across experiments and simulations presented here, our analyses failed to identify situations where use of SCIM would induce a considerable disadvantage compared to reference methods. Methodological differences, e.g., underlying assumptions of the methods and associated numerical effort, also did not negatively affect the range of situations where SCIM is applicable. Under the scenario of *p*-values larger than 0.01 and a simultaneous emphasis on high specificity, the permutation test might be preferred when its high computational cost is irrelevant. However, this scenario is of limited relevance for most studies. We expect that future studies will contribute to a broader understanding of the algorithm's qualities.

### 4.2. Smoothing of Classification Performance Maps

Spatial smoothing influences the outcome of statistical tests and estimated posterior probabilities, since it alters the underlying AUC (and respectively accuracy) distributions in informative and non-informative brain regions in different ways. Regions of high spatial continuity, corresponding to comparably low standard deviation, are expected to coincide with informative regions of high mean AUC and accuracy, respectively. Smoothing further reduces their deviation even more, and thus, reduces the number of searchlights at the far right-hand tail of the distribution, as would be detected by a fixed threshold. Non-informative regions are characterized by lower spatial continuity, resulting in a comparatively larger reduction of the standard deviation being induced by spatial smoothing. The proposed SCIM method adaptively tracks changes in these underlying distributions since the two-component Gaussian mixture model adapts to the informative and non-informative distributions that are implied by the observed data, i.e., smoothed or unsmoothed AUC and accuracy. Spatial smoothing applied to the random permutation and binomial reference methods has predominantly the effect of reducing spatially “noisy” false positive searchlights (cf. Figures 3, 4, third and fourth rows), however, occasionally coinciding with a reduction in informative map extent. The higher sensitivity that can be achieved with all three methods is also reflected in the panels A and B of Figure 6 and the ROC curves in panel F of the same figure. The SCIM method benefits from smoothing in particular through the inclusion of additional searchlight volumes into the informative region estimate, with an overall increase in physiological plausibility (cf. Figures 3, 4, second row).

### 4.3. Classification Performance Measure

Group result maps obtained from SCIM analysis in Figures 13A,B display larger informative regions resulting from the AUC measure than from the accuracy measure, with the former providing a better match across the spatial extent of physiologically task-relevant areas known from literature (cf. discussion of physiological results below). Robustness, evaluated as the dependency of the number of informative searchlights on applied thresholds, shown in Figure 9, is comparable for both performance measures in the SCIM method. For random permutation tests, results based on accuracy are hard to interpret and inconsistent with informative regions known from literature for the task of semantic processing.

These observations likely reflect principled advantages of the AUC measure for classifier evaluation over the accuracy measure. AUC has been shown to provide a performance measure that is invariant to a priori class probabilities and exhibits increased sensitivity and decreased standard error (Green and Swets, 1966; Spackman, 1989; Bradley, 1997). In the context of the SCIM method, it provides us with a tool to perform reliable regularization and model selection in the SVM learning step and, thus, prevents over-fitting in the searchlight classification step. The informative and non-informative distributions, obtained in the GMM step of the SCIM method, are characterized by a small standard deviation due to effectively “decreased noise” in the AUC measure. This facilitates the decomposition into an informative and non-informative searchlight distribution in the SCIM method and increases the robustness of informative regions in IRMs.

### 4.4. Physiological Results

The analyses presented above consistently found informative brain regions in auditory cortex areas that are associated with speech perception. Specifically, we identified superior temporal sulcus (STS) which was shown to play a role in speech processing in general (Uppenkamp et al., 2006; Osnes et al., 2011; Markiewicz and Bohland, 2016) and the processing of intelligible speech in particular (Davis and Johnsrude, 2003; Abrams et al., 2012; McGettigan et al., 2012). Heschl's gyrus, another brain region labeled as informative by the SCIM method, has previously been connected with different degrees of speech clarity (Wild et al., 2012), perception of vowels (Formisano et al., 2008), intelligible speech (McGettigan et al., 2012), and syllables (Markiewicz and Bohland, 2016). In auditory cortical areas associated with higher order auditory processing, regions in inferior frontal sulcus showed informative content for the semantic vs. non-semantic speech contrast. They had previously been reported to be relevant for semantic and phonological processing, word and syllable counting (Poldrack et al., 1999), as well as for hierarchical structures and sentence processing (Makuuchi et al., 2009), speech working memory (Friederici et al., 2006), and processing of intelligible speech (Abrams et al., 2012). In fronto-cortical areas, our group analyses showed reliable results in cingulate gyrus (*p*_SCIM_ < 0.001), which is consistent with findings by Adank and Devlin (2010) for processing of auditory sentences, and for output-related vowel information by Markiewicz and Bohland (2016). In Rissmann et al. (2003), this region showed higher activation for words compared to non-words. Binder et al. (2009) described this area in their meta-analysis as interface between the semantic retrieval and episodic encoding systems.

## 5. Conclusion

This work explored a novel method to evaluate neurophysiological data that was tested on data obtained from an auditory fMRI study, investigating cognitive processes during the semantic processing of speech. The method is based on searchlight classification analysis with subsequent division of searchlight results into informative and non-informative searchlight regions and permits a more robust discrimination of informative vs. non-informative cortical regions than common evaluation methods like random permutation tests or the binomial test. Informative regions obtained with the method are qualitatively consistent with those obtained from reference methods. Yet, *a posteriori* probabilities resulting from the SCIM method dissociate into two distinctly separate distributions, whereas separation of significant from non-significant results in the reference methods are highly threshold-dependent. Since changes in applied thresholds change resulting informative region maps to a lesser degree, the SCIM method provides an evaluation tool that increases the specificity of multivariate fMRI analysis without degrading sensitivity in a considerable manner. The method is applicable to all fMRI studies that permit a classification of BOLD responses into distinct classes of tasks or conditions. It is beneficial in particular for fMRI studies in which sparse imaging is used and the data-set is rather small. The example data presented in this study illustrate that the procedure allows for robust identification of plausible group effects that were not found with univariate statistical analysis.

## Data Availability Statement

The raw data supporting the conclusions of this article will be made available by the authors, without undue reservation.

## Ethics Statement

The studies involving human participants were reviewed and approved by EK-2010/06/21, DFG Application UP 10/2-2. The patients/participants provided their written informed consent to participate in this study.

## Author Contributions

SU contributed the fMRI experiment design. AU and SU acquired the fMRI data. AU and JA analyzed the fMRI data and developed the proposed method. AU implemented the algorithms and analyses, and wrote the initial manuscript. JA proposed the GMM and *a-posteriori* model and wrote sections of the manuscript. All authors contributed to manuscript revision, read, and approved the submitted version.

## Conflict of Interest

The authors declare that the research was conducted in the absence of any commercial or financial relationships that could be construed as a potential conflict of interest.
