# Supplementary material for: Searchlight Classification Informative Region Mixture Model (SCIM): Identification of Cortical Regions Showing Discriminable BOLD Patterns in Event-Related Auditory fMRI Data
Source: Front Neurosci. 2021 Feb 1;14:616906. doi: 10.3389/fnins.2020.616906 (PMC7882477; doi:10.3389/fnins.2020.616906)
Supplement: Supplementary file 1 [file Data_Sheet_1.PDF]

Supplementary Material

0.1 Figures

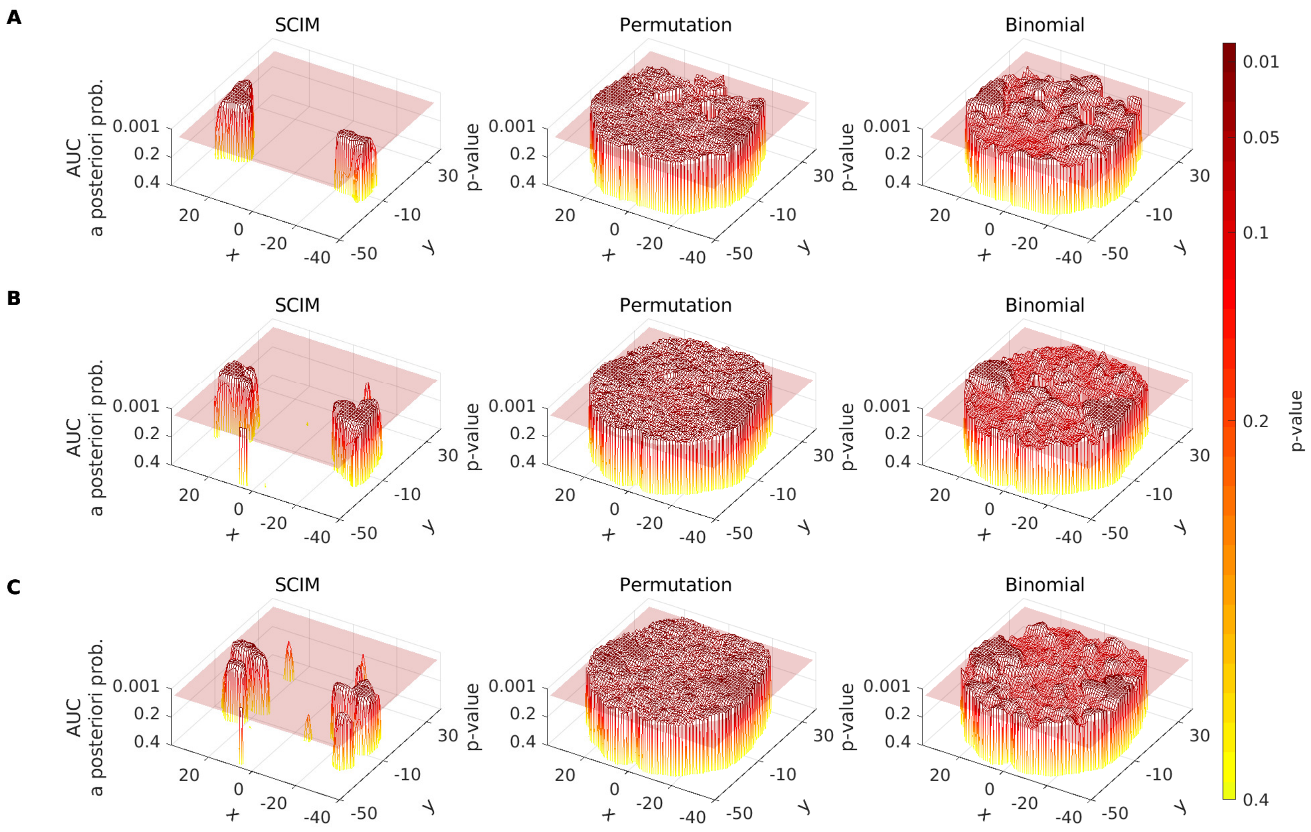

Figure S1. Spatial distribution of a-posteriori probabilities p_{SCIM} (SCIM) and p-values (random permutationtest and binomial test) across a single slice (z= -2mm, single subject, evaluation measure AUC, spatialsmoothing for SCIM, random permutation and binomial) of single subject results from three different subjects. Panel A depicts results for subject 1, panel B depicts results for subject 2 and panel C depicts results for subject 3.

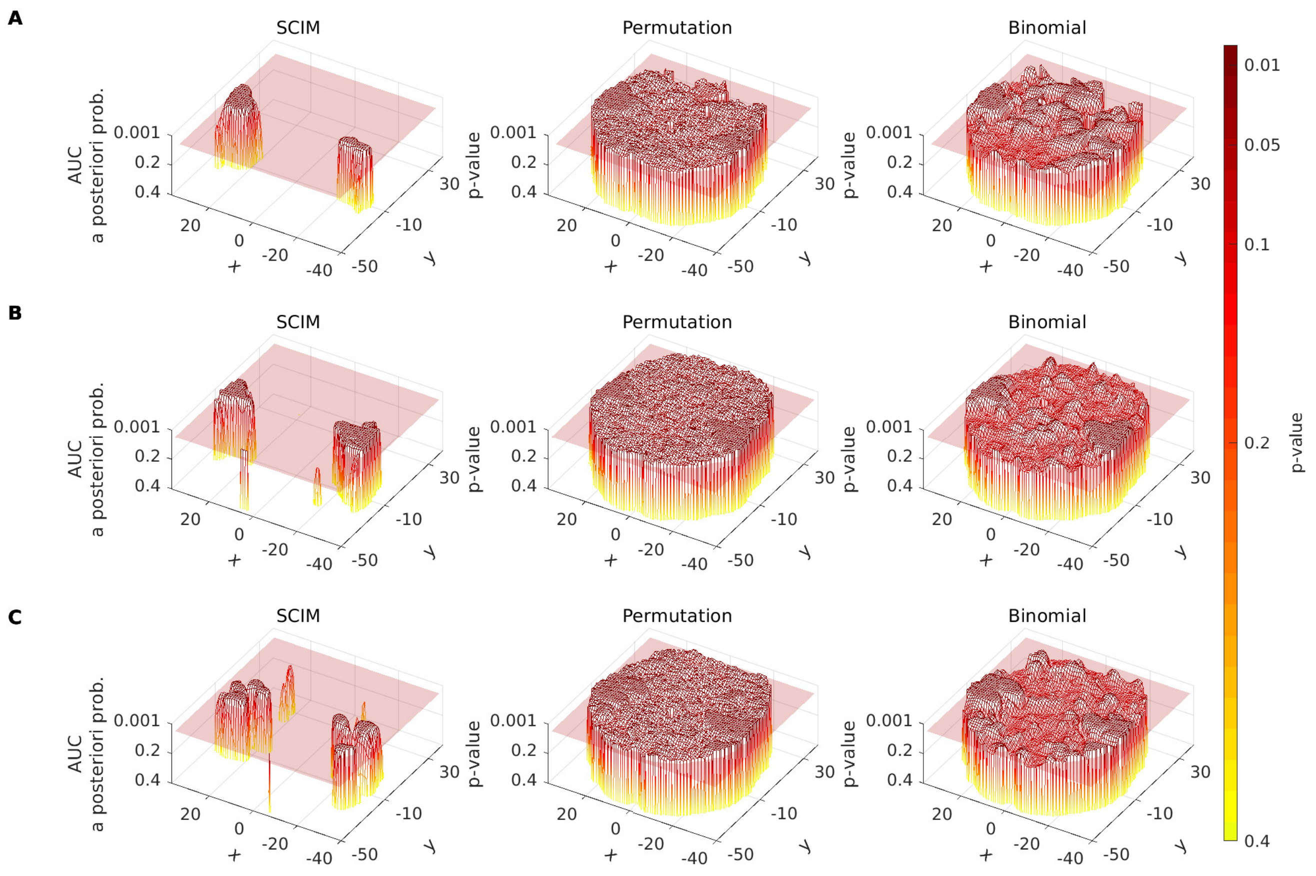

Figure S2. Spatial distribution of a-posteriori probabilities p_{SCIM} (SCIM) and p-values (random permutationtest and binomial test) across a single slice (z= 2mm, single subject, evaluation measure AUC, spatialsmoothing for SCIM, random permutation and binomial) of single subject results from three different subjects. Panel A depicts results for subject 1, panel B depicts results for subject 2 and panel C depicts results for subject 3.

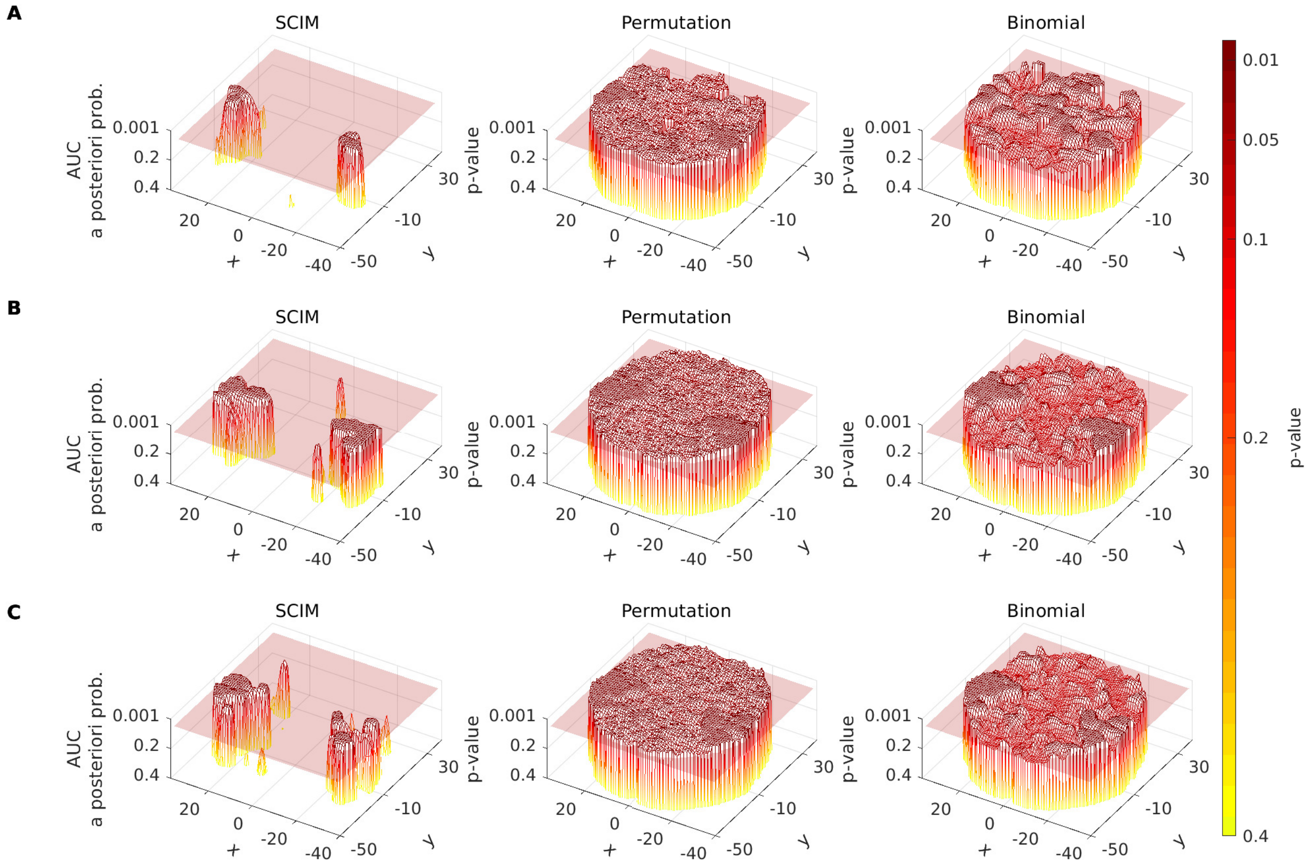

Figure S3. Spatial distribution of a-posteriori probabilities p_{SCIM} (SCIM) and p-values (random permutationtest and binomial test) across a single slice (z= 10mm, single subject, evaluation measure AUC, spatial smoothing for SCIM, random permutation and binomial) of single subject results from three different subjects. Panel A depicts results for subject 1, panel B depicts results for subject 2 and panel C depicts results for subject 3.

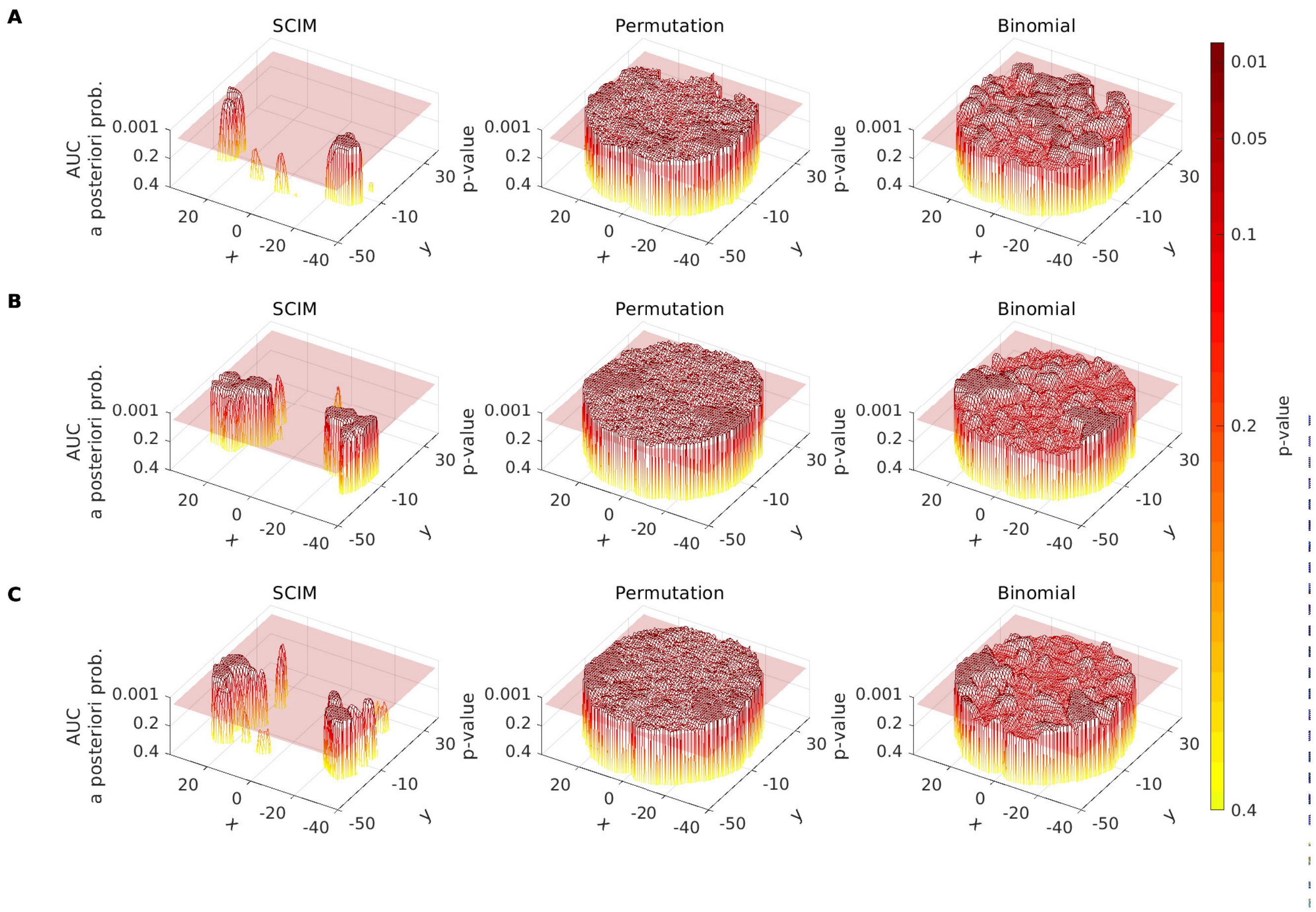

Figure S4. Spatial distribution of a-posteriori probabilities p_{SCIM} (SCIM) and p-values (random permutationtest and binomial test) across a single slice (z= 14mm, single subject, evaluation measure AUC, spatial smoothing for SCIM, random permutation and binomial) of single subject results from three different subjects. Panel A depicts results for subject 1, panel B depicts results for subject 2 and panel C depicts results for subject 3.

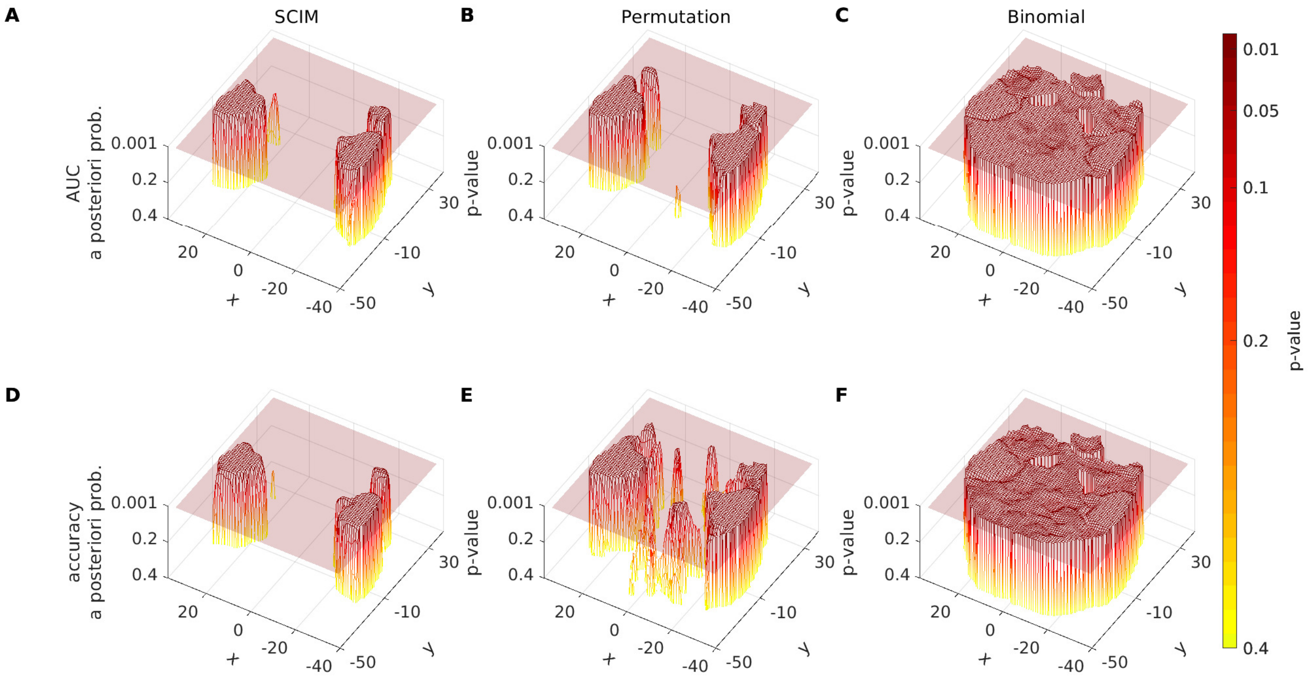

Figure S5. Spatial distribution of a-posteriori probabilities p_{SCIM} (in the panels A and D) and p-values (random permutation test in the panels B and E and binomial test in the panels C and F) across a single slice from group result maps (z= -2mm, group results, evaluation measure AUC (A-C) and accuracy (D-F), spatial smoothing.

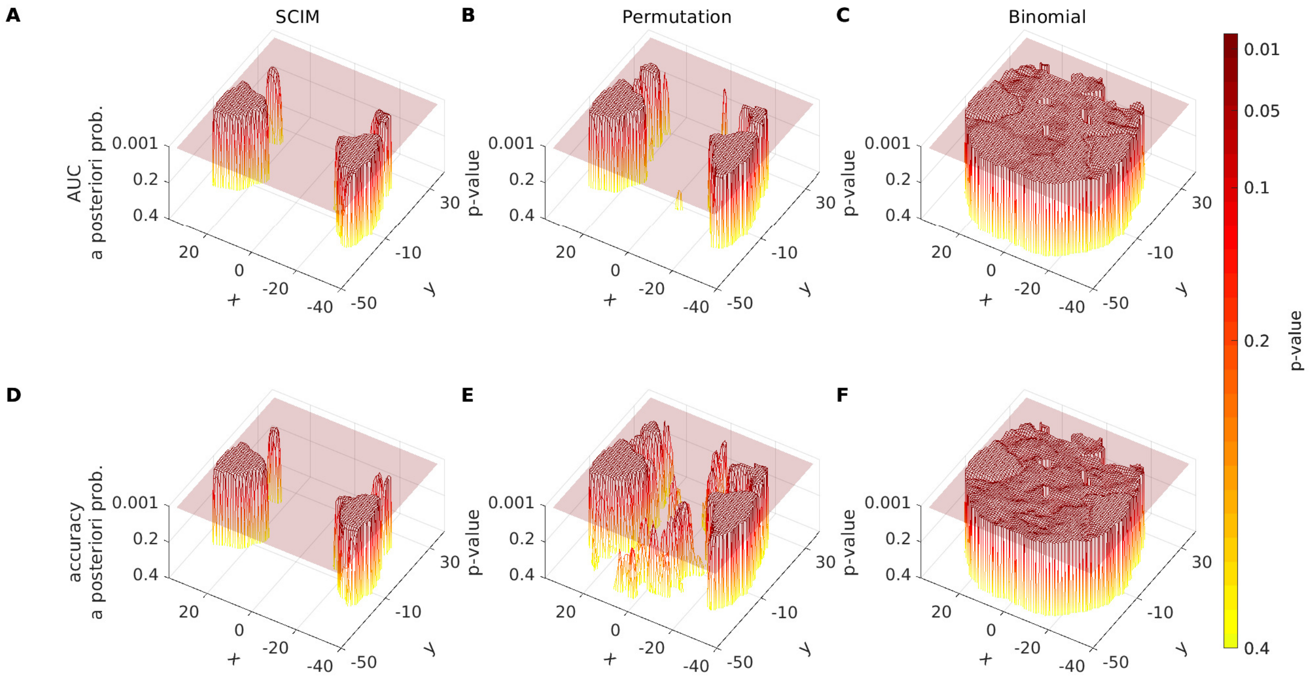

Figure S6. Spatial distribution of a-posteriori probabilities p_{SCIM} (in the panels A and D) and p-values (random permutation test in the panels B and E and binomial test in the panels C and F) across a single slice from group result maps (z= 2mm, group results, evaluation measure AUC (A-C) and accuracy (D-F), spatial smoothing.

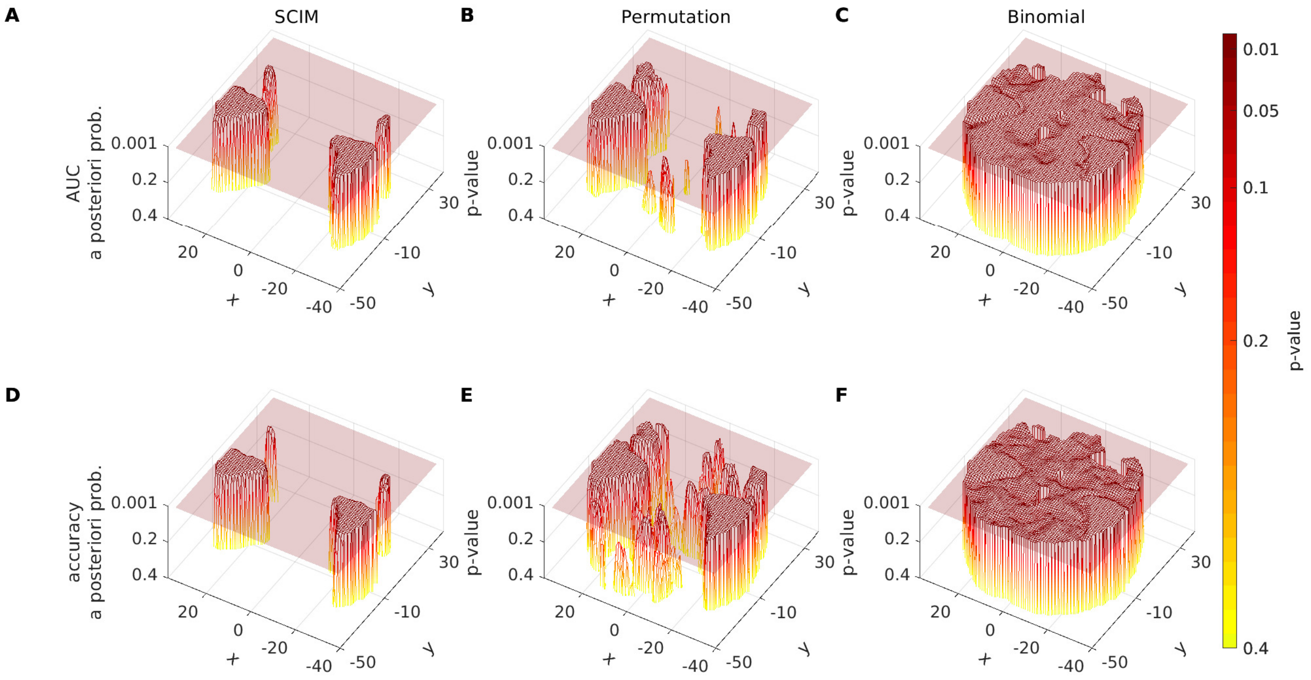

Figure S7. Spatial distribution of a-posteriori probabilities p_{SCIM} (in the panels A and D) and p-values (random permutation test in the panels B and E and binomial test in the panels C and F) across a single slice from group result maps (z= 10mm, group results, evaluation measure AUC (A-C) and accuracy (D-F), spatial smoothing.

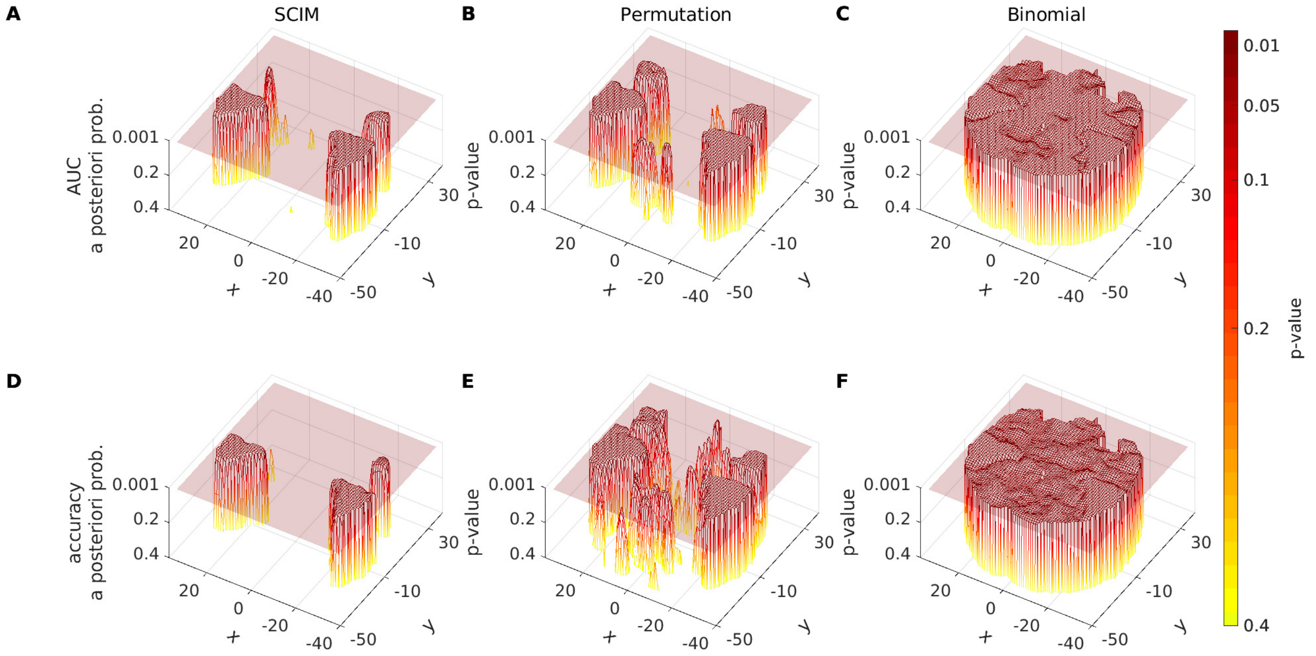

Figure S8. Spatial distribution of a-posteriori probabilities p_{SCIM} (in the panels A and D) and p-values (random permutation test in the panels B and E and binomial test in the panels C and F) across a single slice from group result maps (z= 14mm, group results, evaluation measure AUC (A-C) and accuracy (D-F), spatial smoothing.

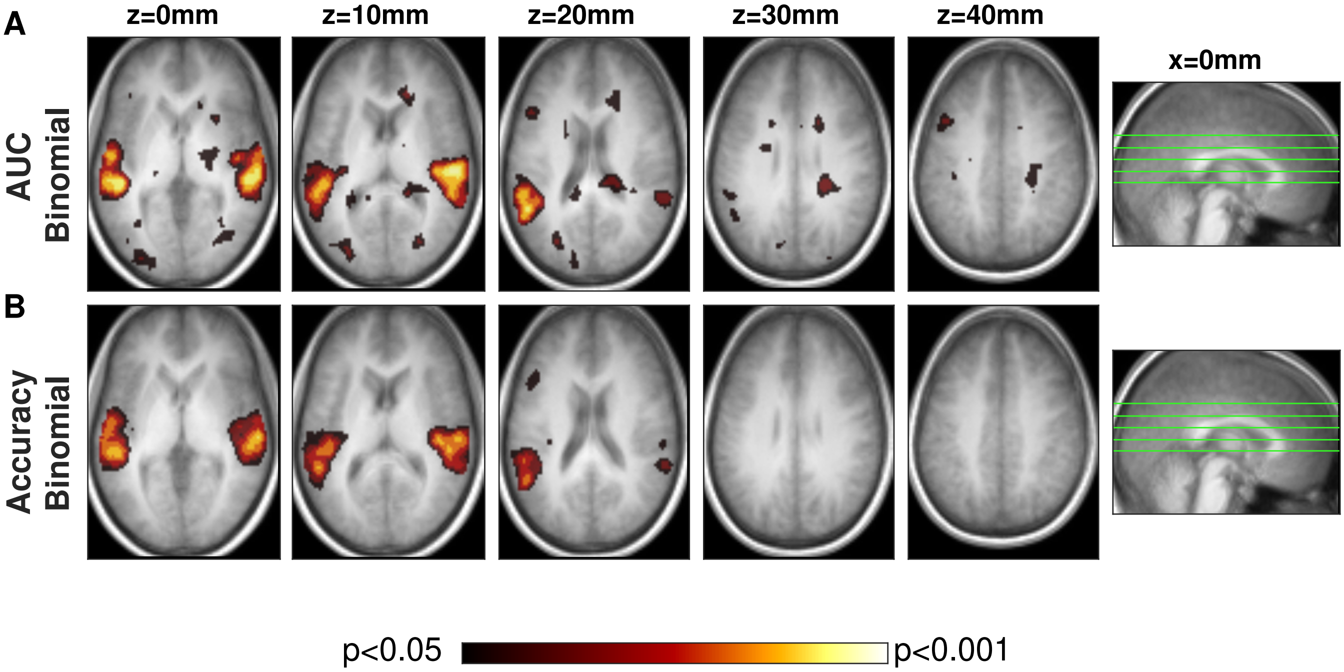

Figure S9. Group result maps for the contrast semantic speech vs. non-semantic speech, with binomial test on (A) AUC maps and (B) accuracy maps in five transversal slices and one saggital slice to display location of transversal slices.
